# The Histidine Ammonia Lyase of Trypanosoma cruzi Is Involved in Acidocalcisome Alkalinization and Is Essential for Survival under Starvation Conditions

**DOI:** 10.1128/mBio.01981-21

**Published:** 2021-11-02

**Authors:** Brian S. Mantilla, Cristina Azevedo, Paul W. Denny, Adolfo Saiardi, Roberto Docampo

**Affiliations:** a Center for Tropical and Emerging Global Diseases, University of Georgia, Athens, Georgia, USA; b Department of Biosciences, Durham University, Durham, United Kingdom; c Medical Research Council Laboratory for Molecular Cell Biology, University College of London, London, United Kingdom; d InnovPlantProject Collaborative Laboratory, Department of New Biopesticides, Elvas, Portugal; e Department of Cellular Biology, University of Georgia, Athens, Georgia, USA; Harvard T. H. Chan School of Public Health

**Keywords:** acidocalcisome, alkalinization, histidine ammonia lyase, polyphosphate, *Trypanosoma cruzi*

## Abstract

Trypanosoma cruzi, the agent of Chagas disease, accumulates polyphosphate (polyP) and Ca^2+^ inside acidocalcisomes. The alkalinization of this organelle stimulates polyP hydrolysis and Ca^2+^ release. Here, we report that histidine ammonia lyase (HAL), an enzyme that catalyzes histidine deamination with production of ammonia (NH_3_) and urocanate, is responsible for acidocalcisome alkalinization. Histidine addition to live parasites expressing HAL fused to the pH-sensitive emission biosensor green fluorescent protein (GFP) variant pHluorin induced alkalinization of acidocalcisomes. PolyP decreased HAL activity of epimastigote lysates or the recombinant protein but did not cause its polyphosphorylation, as determined by the lack of HAL electrophoretic shift on NuPAGE gels using both *in vitro* and *in vivo* conditions. We demonstrate that HAL binds strongly to polyP and localizes to the acidocalcisomes and cytosol of the parasite. Four lysine residues localized in the HAL C-terminal region are instrumental for its polyP binding, its inhibition by polyP, its function inside acidocalcisomes, and parasite survival under starvation conditions. Expression of HAL in yeast deficient in polyP degradation decreased cell fitness. This effect was enhanced by histidine and decreased when the lysine-rich C-terminal region was deleted. In conclusion, this study highlights a mechanism for stimulation of acidocalcisome alkalinization linked to amino acid metabolism.

## INTRODUCTION

Trypanosoma cruzi, the etiologic agent of Chagas disease, is characterized by the presence of acidocalcisomes, lysosome-related organelles containing large amounts of polyphosphate (polyP) bound to organic cations, such as basic amino acids and polyamines, and inorganic cations like calcium, magnesium, potassium, sodium, and zinc (1). These organelles are acidified by two proton pumps, a vacuolar H^+^-ATPase (V-ATPase) (2) and a vacuolar H^+^ pyrophosphatase (VP1) (3), and possess a P-type Ca^2+^-ATPase for Ca^2+^ uptake (4) and an inositol 1,4,5-trisphosphate receptor (IP_3_R) for Ca^2+^ release (5). Previous work (6) demonstrated that alkalinization of acidocalcisomes by incubation of the parasites in the presence of a combination of ionomycin and nigericin or NH_4_Cl was followed by polyP hydrolysis and Ca^2+^ release, probably as a consequence of the stimulation of an acidocalcisome exopolyphosphatase that has increased activity at neutral or alkaline pH.

PolyP hydrolysis (6), cytosolic Ca^2+^ increase (7), and alkalinization of acidocalcisomes (8) also occur when cells are subjected to hypo-osmotic stress. Under these conditions, an increase in intracellular ammonium (NH_4_^+^) due to amino acid catabolism was proposed to have a role in acidocalcisome alkalinization (8). In addition, electrophysiological experiments with DT-40-3KO cells expressing a trypanosome IP_3_R in their nuclear/endoplasmic reticulum membrane found that luminal orthophosphate (P_i_) or pyrophosphate (PP_i_), the hydrolysis products of polyP, and neutral or alkaline pH can stimulate IP_3_-generated currents, while polyP_3_, the polymeric polyP of acidocalcisomes, inhibits these currents (9). Taken together, these results suggest that alkalinization by ionophores or NH_4_^+^ production stimulates an acidocalcisome exopolyphosphatase activity, resulting in hydrolysis of polyP with the formation of P_i_ and PP_i_ and in release of Ca^2+^ and other cations bound to polyP. P_i_ and PP_i_ then stimulate the luminal region of the IP_3_-stimulated receptor, increasing its open probability and thus releasing Ca^2+^ into the cytosol. However, the physiological mechanisms involved in acidocalcisome alkalinization are still unknown.

The use of biotinylated polyP to identify polyP-binding proteins (polyP-ome) in lysates from T. cruzi epimastigotes allowed the identification of >25 putative polyP-interacting proteins (10). The most abundant of these was, by far, the histidine ammonia-lyase (HAL) (10). HAL catalyzes the conversion of histidine into urocanate and ammonia (NH_3_), which is protonated to ammonium (NH_4_^+^; pK_a_, 9.25) at physiological pH. This enzyme is developmentally regulated and highly expressed in epimastigotes (11).

Here, we report that HAL has cytosolic and acidocalcisome localization and that it is able to deaminate histidine inside the organelle, as demonstrated by alkalinization upon addition of histidine to live parasites labeled with a pH biosensor-tagged enzyme. In agreement with these results, addition of histidine to digitonin-permeabilized parasites or isolated acidocalcisomes stimulates H^+^ release. HAL binds to polyP, which inhibits its enzymatic activity, but this interaction is mediated by an electrostatic interaction rather than by lysine polyphosphorylation. Expression of HAL in yeast devoid of endopolyphosphatases was toxic to the cells. We identified a C-terminal lysine-rich region in HAL whose deletion inhibits epimastigote growth in starvation medium supplemented with histidine as an energy source and which reverses its toxicity when expressed in yeast.

## RESULTS

### HAL localizes to the acidocalcisomes of T. cruzi.

**FIG 1:**
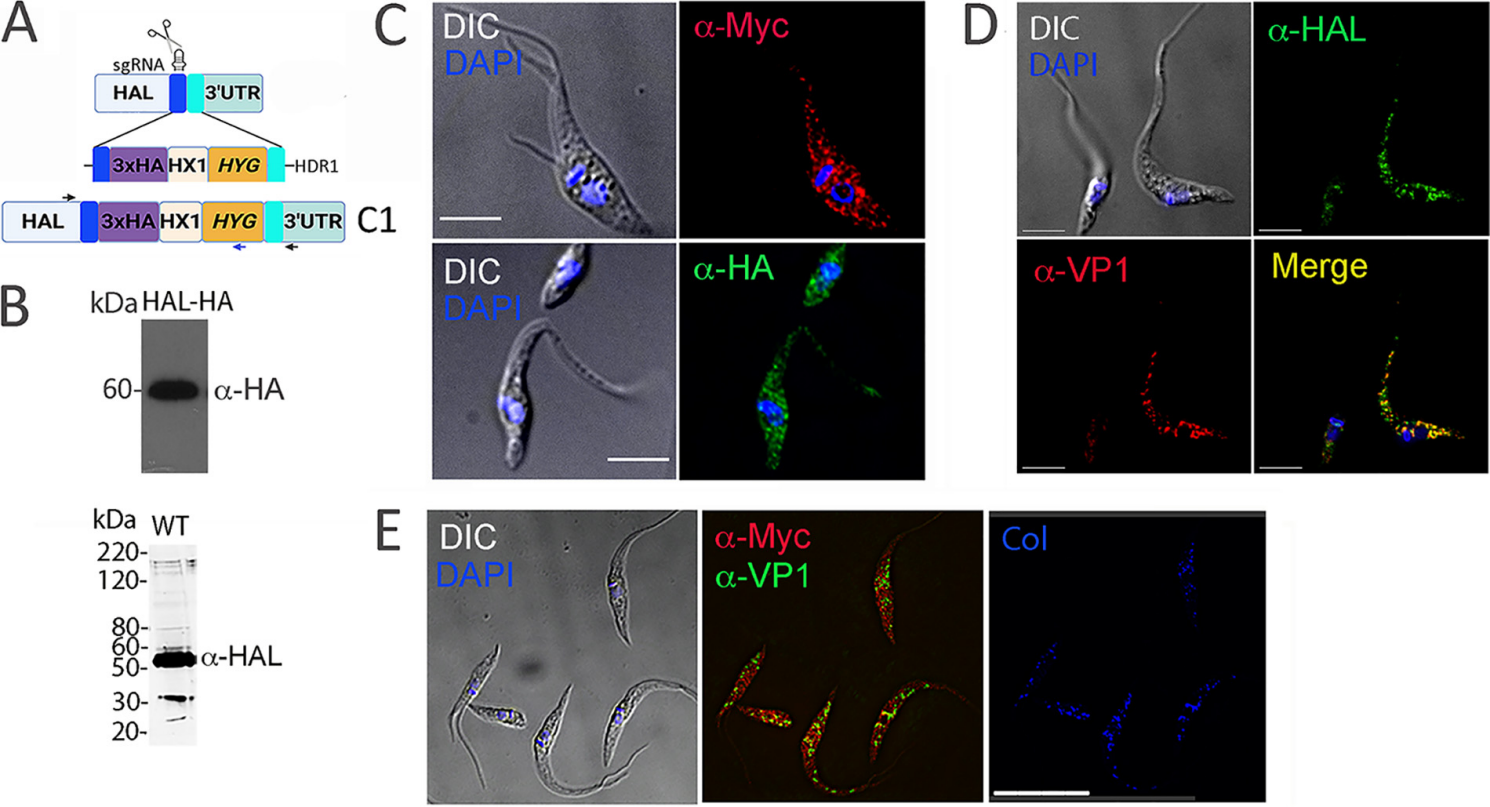
Subcellular localization of HAL in Trypanosoma cruzi. (A) Schematic illustration of CRISPR/Cas9-mediated endogenous tagging. A HAL-specific sgRNA was used to target the insertion of a donor template (1.4 kb) flanked by homology arms (100 nt) and harboring a 3×HA tag sequence to be expressed in frame with HAL. An additional cell line using the 3×c-Myc tag was also generated. The intergenic region (*HX1*) drives the expression of the hygromycin resistance gene (*HYG*), used as a selection marker. Specific primers were designed for validation of cell lines (arrows). This cell line was named C1. (B) Western blot analyses of epimastigote lysates expressing HAL-HA using anti-HA antibodies (top) or WT parasite lysates using anti-HAL antibodies (bottom). (C) Immunofluorescence assays of HAL-tagged epimastigotes using anti-HA (green) or anti-c-Myc (red) antibodies. DAPI was used for DNA staining and is shown merged with the differential interference contrast (DIC) image. Bars = 5 μm. (D) Immunofluorescence assay using anti-VP1 (red) that labels the acidocalcisomes and mouse anti-HAL polyclonal serum (green). Yellow staining indicates colocalization region in wild-type (WT) epimastigotes. (E) Immunofluorescence analysis of HAL in c-Myc-tagged parasites using anti-c-Myc and anti-VP1 antibodies. The blue panel shows the colocalization (Col) areas obtained from this labeling supported by a Pearson coefficient of colocalization of 0.6 ± 0.04. This panel was generated after deconvolution iterations and simultaneous analysis of green/red channel fluorescence. Bar = 15 μm.

HAL was endogenously tagged with 3×c-Myc and 3×HA using CRISPR-Cas9 genome editing (Fig. 1A). The protein was detected in parasite lysates by Western blot analysis, which showed a band consistent with its predicted molecular mass (59.5 kDa) (Fig. 1B, top). Immunofluorescence analysis of epimastigote forms using the two C-terminal tags revealed a cytosolic distribution with some large puncta (Fig. 1C). Antibody detection of HAL using polyclonal serum raised against this protein (Fig. 1B, bottom) revealed a similar labeling pattern (Fig. 1D). To determine whether these puncta correspond to acidocalcisomes, we performed colocalization studies. Untagged HAL colocalized with the vacuolar H^+^-pyrophosphatase (TcVP1) (Fig. 1D), an acidocalcisome marker (1). A similar result was obtained by colocalization of TcVP1 and c-Myc-tagged HAL (Fig. 1E).

**FIG 2:**
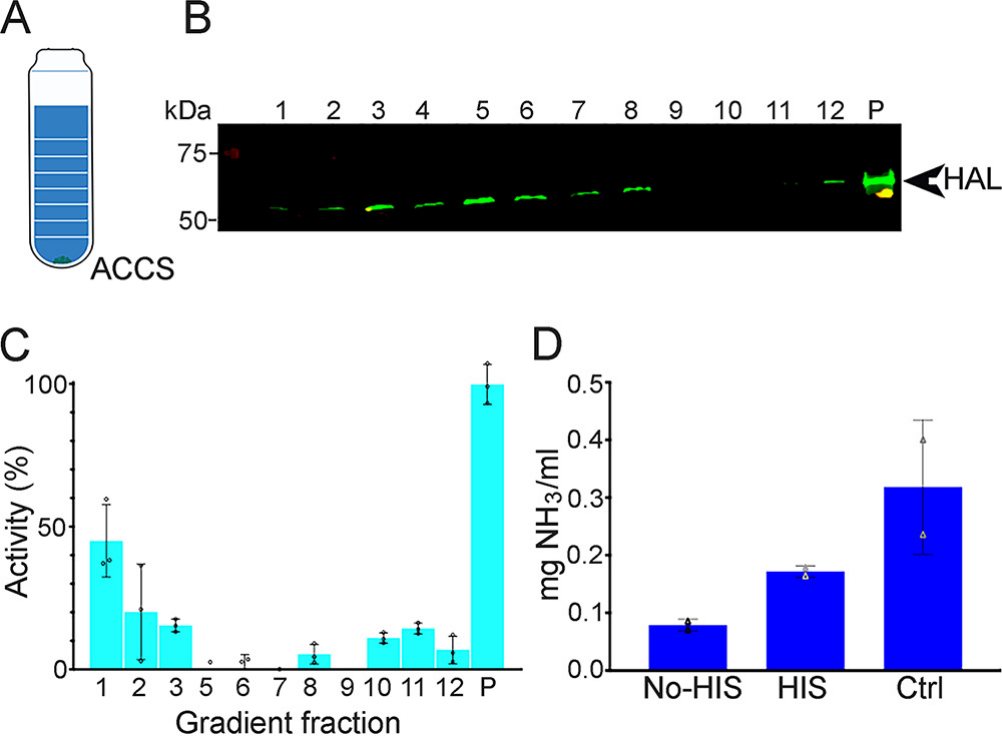
Detection of HAL and ammonia in acidocalcisome fractions. (A) Cellular fractionation was performed using a density gradient of iodixanol where acidocalcisomes (ACCS) are present in the pellet fraction. (B) Western blot analysis of the 12 subcellular fractions (1 ml each) and pellet (P) using a mouse polyclonal anti-HAL antibody. The expected molecular monomeric size for HAL is 59 kDa. (C) Fractions and pellet were also analyzed for TcVP1 activity (measured as AMDP-sensitive P_i_ release). The *y* axis indicates relative distribution; the *x* axis indicates fraction number; the data are means and standard deviations (SD) (as a percentage of the total recovered activity) from two independent experiments (*n* = 3). (D) The pellet fraction containing HAL and acidocalcisomes was used to detect ammonia produced without (No His) or with (HIS) l-histidine (5 mM). A control (Ctrl) with glutamate/glutamate dehydrogenase was used as a positive control as indicated by the manufacturer. Values are means ± SD from two independent experiments.

To confirm the localization of HAL in acidocalcisomes, we isolated the organelles by differential centrifugation followed by density gradient ultracentrifugation using iodixanol, as described in Materials and Methods. Figure 2A shows a scheme of the iodixanol gradient where acidocalcisomes are recovered in the precipitate. Western blot analysis of the fractions using HAL antibody (Fig. 2B) and distribution of the acidocalcisome marker (aminomethylenediphosphonate [AMDP]-sensitive TcVP1) in the gradient (Fig. 2C) showed enrichment of HAL and TcVP1 in the pellet. To verify if HAL enzymatic activity was present in the pellet fraction, this was incubated with histidine, and the formation of ammonia was detected (Fig. 2D).

### Histidine-evoked alkalinization of acidocalcisomes.

**FIG 3:**
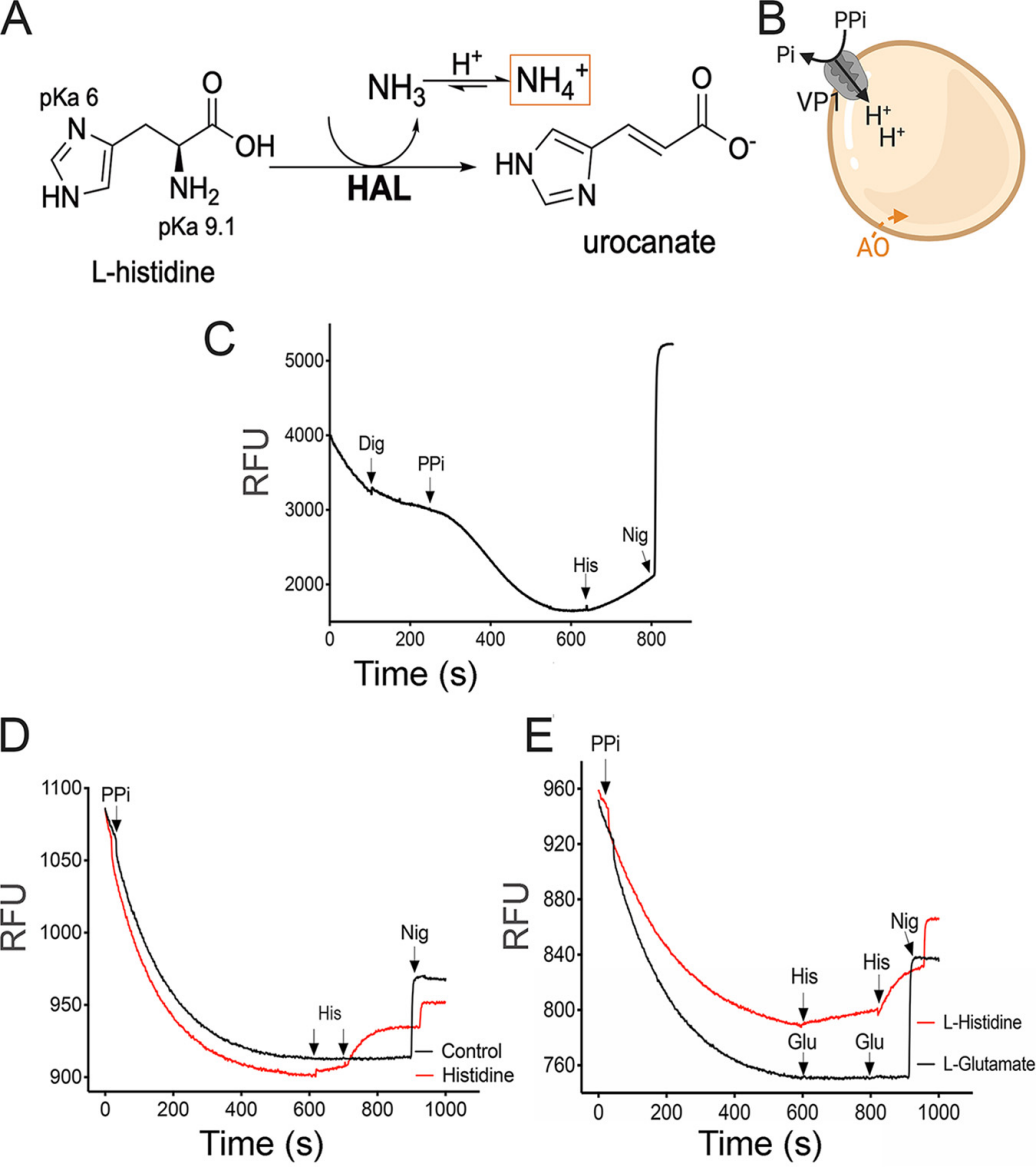
Histidine-evoked alkalinization of acidocalcisomes. (A) The α-amino group of histidine can be deaminated by histidine ammonia lyase (HAL; EC 4.3.1.3), producing urocanate and ammonia. At physiological pH, the equilibrium favors its protonated form, ammonium. (B) Pyrophosphate-driven acidification is mediated by the vacuolar H^+^-PPase (VP1). Acridine orange (AO; 3 μM) accumulates in acidic stores, and fluorescence changes can be followed in digitonin-permeabilized parasites. (C) AO fluorescence measurements in intact epimastigotes permeabilized with digitonin (Dig) followed by sequential additions of 0.1 mM K-pyrophosphate (PP_i_), 5 mM l-histidine (His) and the ionophore nigericin (1 μM Nig). (D) AO fluorescence measurements in acidocalcisomes isolated from epimastigotes. Two pulses of l-histidine were done and compared with the solution used to dissolve l-histidine (control) or 5 mM sodium glutamate (Glu) (E) used as a control. Traces are representative of three independent experiments.

Histidine is a cationic amino acid that can be converted into glutamate through stepwise conversions of its α-amino and imidazole side chain groups. HAL-catalyzed deamination forms urocanate and ammonia (Fig. 3A). We hypothesized that the presence of HAL in acidocalcisomes would lead to the production of ammonia with concomitant H^+^ uptake to form NH_4_^+^ and an increase in organellar pH. Pyrophosphate-driven proton uptake into acidocalcisomes can be monitored *in situ* by using acridine orange (AO), which accumulates in these organelles after digitonin permeabilization (Fig. 3B). Figure 3C shows that addition of l-histidine results in release of the accumulated acridine orange that is completed by the K^+^ ionophore nigericin. Similar results were obtained using isolated acidocalcisomes (Fig. 3D). These results were specific for histidine, as addition of glutamate, which can also deaminate to form NH_3_ in a reaction catalyzed by glutamate dehydrogenase, was unable to release the accumulated acridine orange (Fig. 3E).

### Alkalinization of acidocalcisomes by histidine and effect on parasite viability.

**FIG 4:**
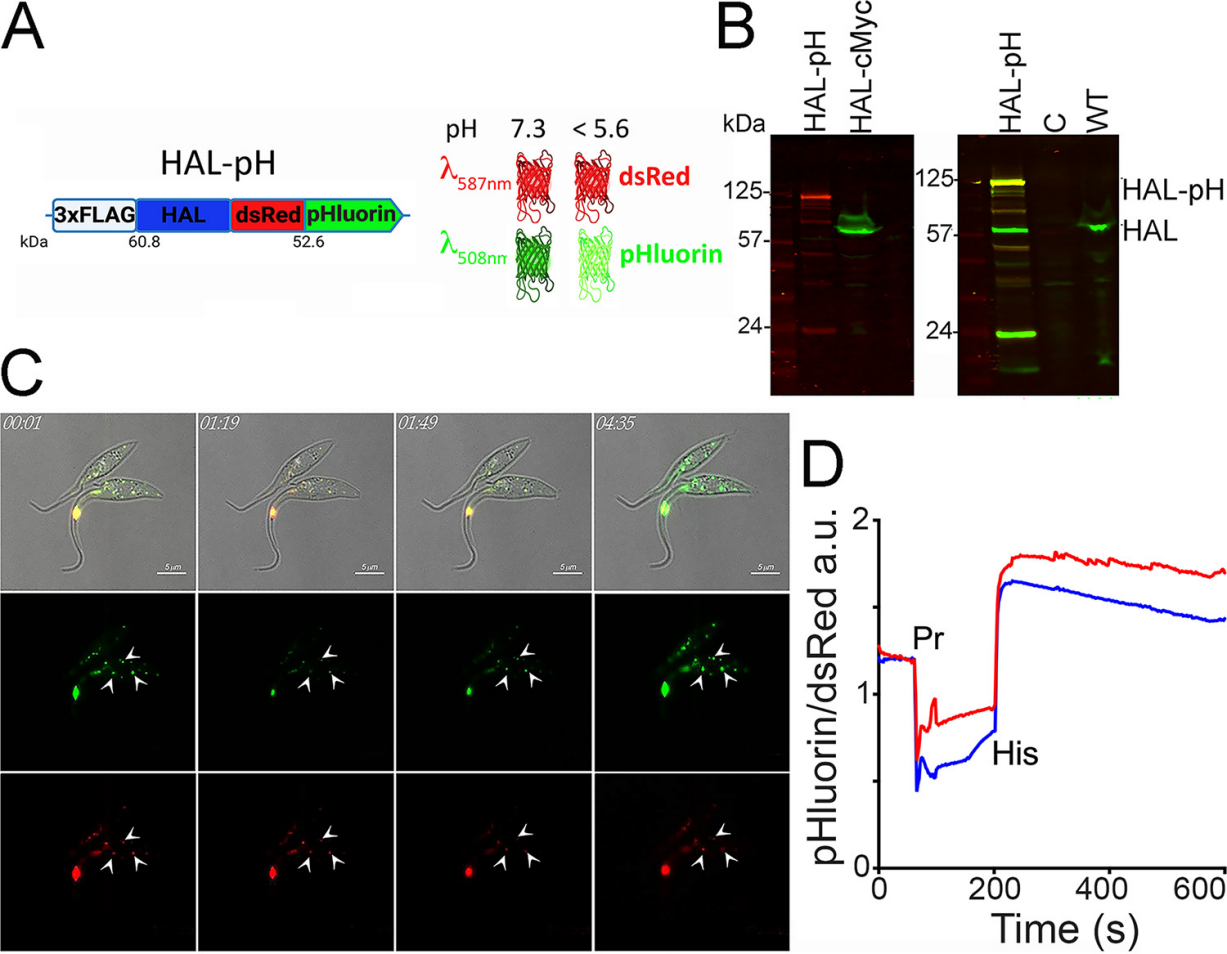
Histidine deamination impact on parasite acidocalcisome pH. (A) Representation of the specific construct harboring an N-terminal tag upstream of the HAL coding sequence followed by two fluorescent proteins in tandem attached to the 3′ extremity of HAL, as indicated. This reporter cassette was termed HAL-pH and contains a neomycin resistance sequence used as selection marker. The green fluorescent pHluorin is quenched at lower pH values (<5.6), whereas the DsRed fluorescence remains bright. The expected molecular mass of this fusion product is ∼114 kDa. (B) Validation of protein expression in HAL-pH-expressing cell lines obtained after drug selection and cell sorting. The expected HAL-pH product was detected using anti-FLAG antibodies, compared to the HAL-c-Myc-tagged protein (∼60 kDa). (Right) Detection of HAL was confirmed with anti-HAL specific antibodies that react against both the native (HAL) and HAL-pH (yellow band) in the same protein sample. C, negative control; WT, wild-type cells. A cross-reacting band (∼30 kDa) of unknown origin was also detected, as in Fig. 1B. (C) Time-lapse recording experiments were done using different substrate additions to the HAL-pH-expressing cells. After 30 s, propionic acid was added, which led to a decrease in pHluorin fluorescence. After 120 s, l-histidine was added, inducing a recovery of intraorganellar pH, as evidenced by an increase in green fluorescence. Frames were extracted from Video S1. Mean fluorescence intensities were recorded in intact cells (00:01), after propionic acid pulse (01:19 to 01:49), and after histidine pulse (04:35). Green and red fluorescence signals correspond to pHluorin and DsRed reporters, respectively. White arrows indicate acidocalcisomes. We show labeling at 01 min 19 s instead of at 40 s to see the change of color of the acidocalcisomes, avoiding the movement due to the addition of propionic acid. (D) Ratiometric analysis of green versus red fluorescence values recorded throughout the experiment (∼600 s) in two different cells, as indicated by red (top) and blue (bottom) lines, in arbitrary units (a.u.). The values were normalized.

To investigate whether alkalinization of acidocalcisomes upon histidine addition occurs in live cells, we transfected epimastigotes with a genetically encoded pH sensor (pHluorin) fused in tandem to the DsRed protein that was tagged at the C terminus of HAL (Fig. 4A). As pHluorin fluorescence is quenched at acidic pH (pH ≤ 5.6) and DsRed is pH insensitive, their simultaneous expression enables the recording of fluorescence changes that are directly associated with alterations in pH, as well as their ratiometric evaluation. We confirmed that correct fusion of this cassette to HAL resulted in a protein product of approximately 115 kDa (termed HAL-pH), as detected by Western blot analysis with anti-FLAG antibody (Fig. 4B, left). When the anti-HAL was used, we observed expression of the endogenous HAL and the HAL-pH fusion product (Fig. 4B, right). Time-lapse recordings of fluorescence changes using these HAL-pH-expressing epimastigotes showed that the intracellular and the acidocalcisome pH can be acidified after a pulse of propionic acid, as shown by the change in fluorescence from yellow (due to the fluorescence of both DsRed and pHluorin) to red (due to the fluorescence of DsRed) (Fig. 4C). Subsequent addition of histidine changed the fluorescence from red to green due to the alkalinization that enhanced pHluorin fluorescence (Fig. 4C; Video S1). Our assays also showed that localization of HAL inside acidocalcisomes was more apparent when pH_i_ was raised after l-histidine or NH_4_Cl addition, as recorded by pHluorin fluorescence (Videos S1 and S2, respectively). No changes in fluorescence were recorded when l-glutamate was added after acidification, showing that this effect was specific for histidine deamination (Video S3). Ratiometric analysis of the fluorescence recordings showed changes in fluorescence upon addition of propionic acid and histidine (Fig. 4D). Notably, a large histidine pulse (16 mM) without previous acidification by propionic acid was lethal, as the cells did not maintain their integrity (Video S4). These data suggest that HAL-catalyzed histidine deamination could be toxic for the parasites, although we cannot rule out the possibility that tagging of the enzyme could affect the enzyme activity/expression within the cells.

10.1128/mBio.01981-21.5 VIDEO S1 Histidine-driven alkalinization after acidic pulse. Live imaging was recorded in parasites seeded onto polylysine coated slides, and changes in fluorescence (DsRed and pHluorin green) were monitored after sequential additions of propionic acid (30 s) followed by l-histidine (1 min 30 s). The assay was followed for approximately 5 min, and the movie was generated using 6 frames per second (fps) at medium speed. Download Movie S1, MOV file, 7.1 MB. Copyright © 2021 Mantilla et al. 2021 Mantilla et al. https://creativecommons.org/licenses/by/4.0/ This content is distributed under the terms of the Creative Commons Attribution 4.0 International license.

10.1128/mBio.01981-21.6 VIDEO S2 Ammonia-driven alkalinization after acidic pulse. Live imaging was recorded in parasites seeded onto polylysine coated slides, and changes in fluorescence (DsRed and pHluorin green) were monitored after sequential additions of propionic acid (30 s) followed by NH_4_Cl (1 min 30 s). The assay was followed for approximately 5 min, and the movie was generated using 6 fps at slow speed. Download Movie S2, MOV file, 9.8 MB. Copyright © 2021 Mantilla et al. 2021 Mantilla et al. https://creativecommons.org/licenses/by/4.0/ This content is distributed under the terms of the Creative Commons Attribution 4.0 International license.

### Interactions of HAL with polyphosphate.

**FIG 5:**
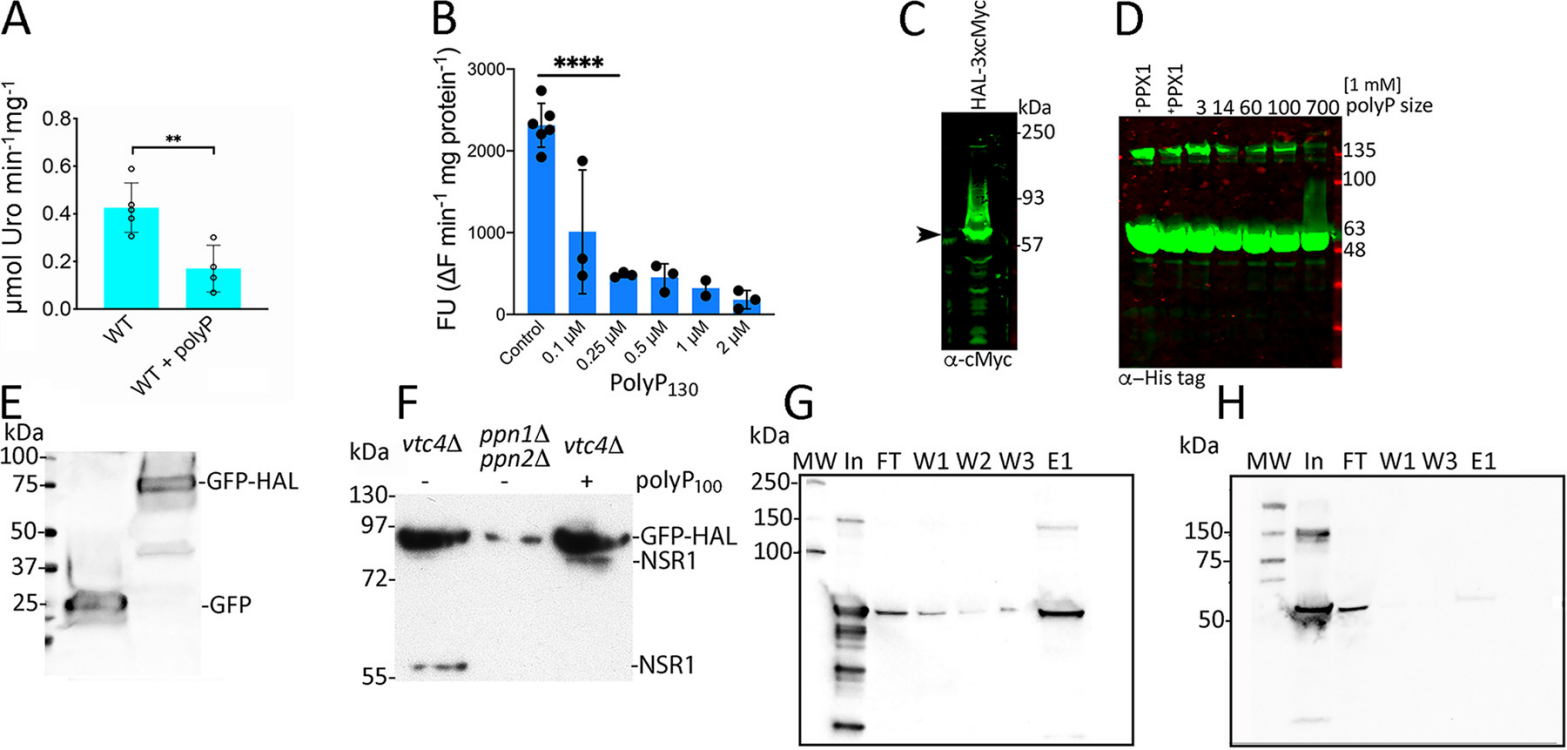
HAL activity is inhibited by polyphosphate but HAL is not polyphosphorylated. (A) The effect of polyP on HAL activity was tested in epimastigote lysates. Values are means ± SEM from four experiments, and differences were compared by using Student's *t* test (**, *P < *0.01). (B) Effect of polyP_130_ on the activity of 6×His-HAL. Bars represent changes in fluorescence obtained in the linear region of histidine conversion under saturating conditions after preincubation (2 min) with polyP. Values are means ± SD of at least three biological replicates and differences were compared by using one-way analysis of variance (ANOVA) (****, *P < *0.0001). (C) Western blot analysis of parasite lysates expressing HAL-c-Myc using anti-c-Myc antibodies. Proteins were resolved by NuPAGE electrophoresis. Arrow shows a band of ∼60 kDa, which is the expected size for HAL. (D) The recombinant 6×His-HAL (rHAL) produced in bacteria was subjected to different conditions to analyze its mobility pattern in NuPAGE electrophoresis and detection with anti-His antibodies. Recombinant HAL was treated with bacterial exopolyphosphatase PPX1 (+) without polyP addition or in the presence of polyP (1 mM in P_i_ units) of various lengths as indicated. Untreated rHAL under normal conditions was used as a control (-PPX1). The mobility for monomeric HAL remained unchanged, and a fainter larger band was also detected. (E) Parental and mutant yeast cells were transformed with HAL protein fused to GFP, and expression was verified by immunoblotting using anti-GFP antibodies. The expected molecular masses are 26.9 and 85 kDa for GFP and GFP-HAL products, respectively. (F) Electrophoretic mobility shift assay of HAL expressed in yeast mutants (*vtc4*Δ and *ppn1*Δ *ppn2*Δ) or expressed in a *vtc4*Δ mutant with or without addition of polyP_100_. Nsr1 (∼55 kDa) was used as a control, as it remains nonpolyphosphorylated when polyP synthesis is absent (*vtc4*Δ) and has decreased mobility (∼91 kDa) after addition of polyP. HAL mobility in the *ppn1*Δ *ppn2*Δ double mutant was similar to that of HAL expressed in *vtc4*Δ mutants. (G and H) Immunoblotting detection of HAL after affinity binding assay with polyP_20_ immobilized in Sepharose beads. Input (In) material corresponds to recombinant protein (10 μg) dissolved in 1 ml of binding buffer. After binding with polyP-beads, unbound material was collected (flowthrough [FT]), and three successive washing steps were performed (W1 to W3). Elution was completed by resuspending the beads in 0.2 ml of buffer containing 1 M NaCl. The monomeric size of HAL is that expected in both the 6×His-HAL (G) and the mutant lacking the last 13 residues of the C terminus of HAL (H).

HAL was the most abundant protein found in the polyP-ome of epimastigote lysates (10), and we therefore investigated the nature of HAL interactions with polyP. First, we studied whether polyP affected HAL activity in epimastigote lysates. HAL activity can be monitored spectrophotometrically following urocanate formation at 277 nm. The addition of 1 mM polyP_100_ (1 mM is the concentration in P_i_ units, or 10 μM when molecular weight [MW] is considered) resulted in a 2-fold decrease in HAL activity (*P* < 0.01) (Fig. 5A). In agreement with these results, we found that polyP_130_ inhibits the activity of the recombinant enzyme in a dose-dependent manner (Fig. 5B).

We then investigated whether this effect could be due to HAL polyphosphorylation. Lysine polyphosphorylation is a nonenzymatic posttranslational modification consisting of the addition of a polyP chain to the lysines in PASK (polyacidic serine and lysine [K]) domains. Polyphosphorylation of proteins is identified by their decrease in mobility (electrophoretic shift) on NuPage gels (12, 13). No apparent changes in mobility were seen when HAL was detected using anti-HAL from T. cruzi lysates (Fig. 5C). As expected, recombinant 6×His-HAL produced in bacteria (Fig. 5D) did not display noticeable shifts in mobility when incubated with Escherichia coli exopolyphosphatase (PPX1), as judged by the size of its monomeric band (∼60 kDa) (Fig. 5D).

To further rule out the polyphosphorylation of HAL, we expressed this protein in yeast deficient in vacuolar polyP, produced by knockout of the catalytic subunit (Vtc4p) of the polyP polymerase vacuolar transporter chaperone complex (VTC) and in yeast deficient in two vacuolar endopolyphosphatases (Ppn1p and Ppn2p) (13). The *vtc4*Δ cells do not make polyP, and therefore, the protein cannot be polyphosphorylated when cells are lysed. The *ppn1*Δ *ppn2*Δ strain lacks the most important yeast enzymes involved in polyP hydrolysis, and therefore, polyP chains bound to released proteins cannot be cleaved in the lysates (14). HAL was fused to enhanced green fluorescent protein (eGFP) at the C terminus and protein detection using anti-GFP confirmed its proper expression in yeast cells (Fig. 5E and Fig. S1A). Unlike for Nsr1 (13), which in the absence of polyP presents a band of ∼55 kDa but when polyP is present shows a band of approximately 95 kDa, no change in the electrophoretic mobility of HAL was detected in either the *vtc4*Δ or the *ppn1*Δ *ppn2*Δ background strains (Fig. 5F). This experiment demonstrates that polyphosphorylation of HAL does not occur endogenously or in the presence of exogenous polyP present upon cell lysis.

10.1128/mBio.01981-21.1 FIG S1 Detection of HAL and PCR diagnostics of knock-in mutant cell lines. (A) Western blot analysis to detect HAL and its mutated version in protein lysates from yeast mutants (*vtc4*Δ and *ppn1*Δ *ppn2*Δ strains). The expected molecular masses are depicted by arrows (GFP-HAL, 85 kDa; GFP, 26.9 kDa). (B) PCR diagnostics was performed using two different set of oligonucleotides that anneal inside the 3′ extremity of HAL and in the 3′ untranslated region outside the recombination site (3′ UTR). An additional reaction using a hygromycin-specific reverse oligonucleotide instead of the 3′ UTR primer was also run (HYG). Arrows depict the amplicon sizes of 1.85 kb (left) and 900 bp (right) amplified in the tagged parasites and the ∼300-bp product, which was amplified only in the intact locus (WT). (C) Western blot analysis of HAL in protein lysates of T. cruzi parental cell line (WT) and HA-tagged cell lines (C1 and C2) resolved by NuPAGE gel electrophoresis. Detection was performed using mouse anti-HAL (1:5,000) or anti-HA (1:2,000) as detailed in Materials and Methods. Arrows indicate monomeric sizes of HAL and HAL-3×HA (∼60 kDa). (D) Effect of polyP_130_ on activity of the 6×His-HAL-C_13_ mutant. Bars represent changes in fluorescence obtained in the linear region of histidine conversion under saturating conditions after preincubation (2 min) with polyP, as indicated in Fig. 5B but using a larger excitation slit width (10 nm). Download FIG S1, TIF file, 2.9 MB. Copyright © 2021 Mantilla et al. 2021 Mantilla et al. https://creativecommons.org/licenses/by/4.0/ This content is distributed under the terms of the Creative Commons Attribution 4.0 International license.

### A positively charged HAL C-terminal region interacts with polyP and is critical for its activity.

Analysis of the HAL primary sequence revealed an intrinsically disordered region (IDR) enriched in lysine residues at its C terminus (last 13 amino acids). IDRs mediate the interaction of proteins with polyanionic molecules such as nucleic acids or phosphate groups (15). To investigate the interaction of HAL with polyphosphate, we performed a protein binding assay using polyP_20_ that was conjugated to polymethacrylate beads through a phosphoramidite linkage. Recombinant 6×His-HAL produced in Escherichia coli cells was incubated with polyP_20_ beads and samples collected at different steps were analyzed by immunoblotting. Interaction of HAL with polyP_20_ was evidenced by its immobilization in the cross-linked polyP_20_ beads. HAL was eluted after increasing the ionic strength (1 M NaCl) in the reaction, as detected using antibody against the His tag (Fig. 5G, lane E1). To assess whether the C terminus of HAL mediates interaction with polyP, we produced a mutated version of HAL lacking this C-terminal region (termed 6×His-HAL-C_13_). PolyP_20_ binding of 6×His-HAL-C_13_ was substantially reduced, as seen in the elution fraction (Fig. 5H).

**FIG 6:**
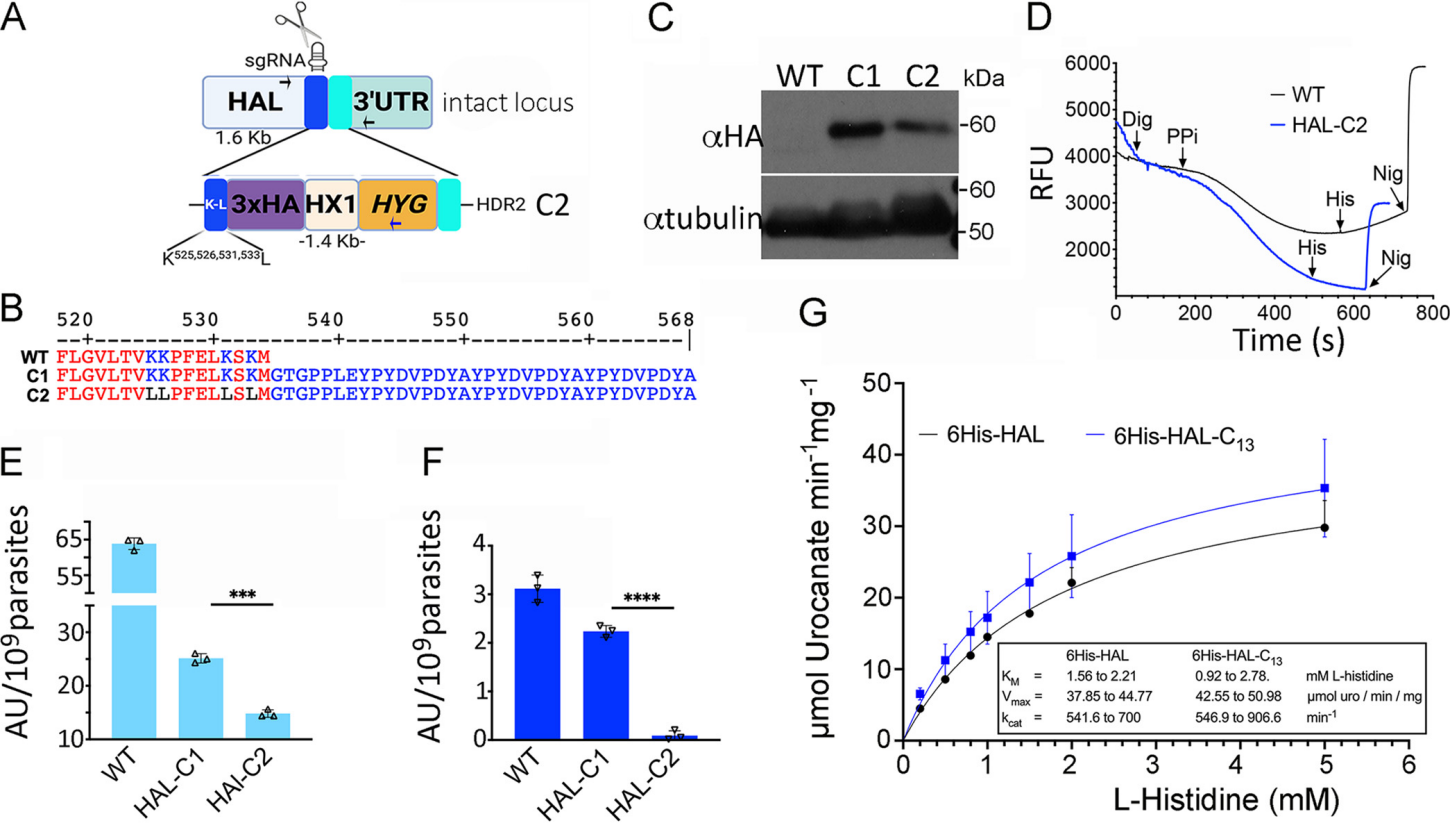
A polylysine motif in HAL has a dominant negative effect in T. cruzi. (A) Schematic representation of the CRISPR/Cas9 strategy used to knock in the HAL gene. The last four lysine residues of HAL were replaced by leucine. This cell line was named C2. Arrows indicate diagnostic primers for PCR. (B) After drug selection and cloning of selected mutants, the amplified PCR products were sequenced using HAL-specific primer where the K525L, K526L, K531L, and K533L mutations were confirmed as well as its in-frame fusion to the 3×HA epitope. Protein alignment was performed using the protein sequence for HAL (UniProtKB no. Q4E133) and those translated from the sequencing data for C1 and C2 parasite cell lines. Only the last 48 residues are shown. (C) Western blot analysis of HAL in 3×HA-tagged parental (WT) and mutant (C1 and C2) parasite cell lines. Anti-α-tubulin (49 kDa) was used as a loading control. (D) Acridine orange experiment using normal and the HAL-C2 mutated cell line. Histidine-driven alkalinization was observed only in WT cells. Other conditions were as for Fig. 3C. (E and F) A viability assay was performed in the C1 and C2 mutant cell lines incubated in normal TAU-3AAG (E) and TAU-histidine media (F) for 48 h. After this time, fluorescence values from the alamarBlue assay were compared and normalized to the number of cells added to each well. Values are means ± SEM from three independent experiments done in triplicate. One-way ANOVA followed by Bonferroni’s statistical test was performed to compare differences found (***, *P* < 0.001; ****, *P < *0.0001). (G) Comparison of enzymatic activities assayed for both 6×His-HAL (black) and the mutated version lacking the cryptic C-terminal site, named 6×His-HAL-C_13_ (blue). Initial velocities were determined with increasing concentrations of l-histidine, and values are means ± SD for biological triplicates. Kinetic parameters were determined by fitting resultant curves to Michaelis-Menten function using the software GraphPad Prism v9 and summarized in the inset (*R*^2^ = 0.99).

To study the role of this IDR in HAL activity and in the parasite viability, we used a CRISPR/Cas9 knock-in strategy to replace the four terminal lysine residues (K525, K526, K530, and K533) with leucine, through the delivery of a donor template containing (C2) or lacking (C1) these mutations (Fig. 6A). Correct insertion into the desired locus was confirmed by PCR (Fig. S1B), targeting the 3′ end of HAL gene and the 3′ untranslated region (UTR) of the hygromycin resistant selection marker. Western blot analyses showed the expression of the proteins (Fig. S1C). The introduced mutations in the HAL-C2 knocked in mutant were confirmed by DNA sequencing of the amplified product from the parasite clones (Fig. 6B). The presence of the 3×HA epitope attached to HAL was also evidenced by sequencing and Western blot analysis with anti-HA antibodies (Fig. 6B and C). To test the effect of the lysine mutation on HAL activity, we measured pyrophosphate-driven AO uptake in permeabilized cells and observed that addition of histidine did not produce alkalinization in HAL-C2 parasites, as occurs with wild-type cells (Fig. 6D, blue trace), suggesting that polyP binding of HAL is important for this activity inside acidocalcisomes. In contrast to the results with cell lysates (Fig. 5A) and the wild-type recombinant protein (Fig. 5B), addition of polyP_130_ was not able to inhibit the activity of the mutated recombinant enzyme (Fig. S1D), supporting the role of the terminal lysines for polyP interaction with HAL.

Histidine has been shown to be very abundant in the hemolymph and excreta of vectors of T. cruzi, such as Rhodnius prolixus (16), and it has been shown that it is one of the amino acids that are crucial for the survival of the parasite under the conditions in the vector (17). Therefore, we compared the viability of epimastigotes in complete triatomine artificial urine (TAU-3AAG), a medium that mimics limited nutritional conditions for the parasite (18). The HAL-C2 mutant parasites showed reduced viability in standard TAU-3AAG medium (Fig. 6E), but incubation in TAU-H medium (with His as the sole carbon source) resulted in parasite death, as measured using alamarBlue (Fig. 6F). Taken together, these data suggest that the C-terminal lysine residues are critical for HAL interaction with polyP and that their mutation leads to an inability of the parasite to metabolize histidine inside acidocalcisomes and to parasite death under nutrient-limiting conditions. Interestingly, the mutation in the C-terminal lysine residues does not affect the enzymatic activity of the recombinant HAL, as judged by the kinetic parameters determined in both isoforms (Fig. 6G, inset).

### HAL expression in yeast confirms a critical role for the lysine-rich C-terminal region.

**FIG 7:**
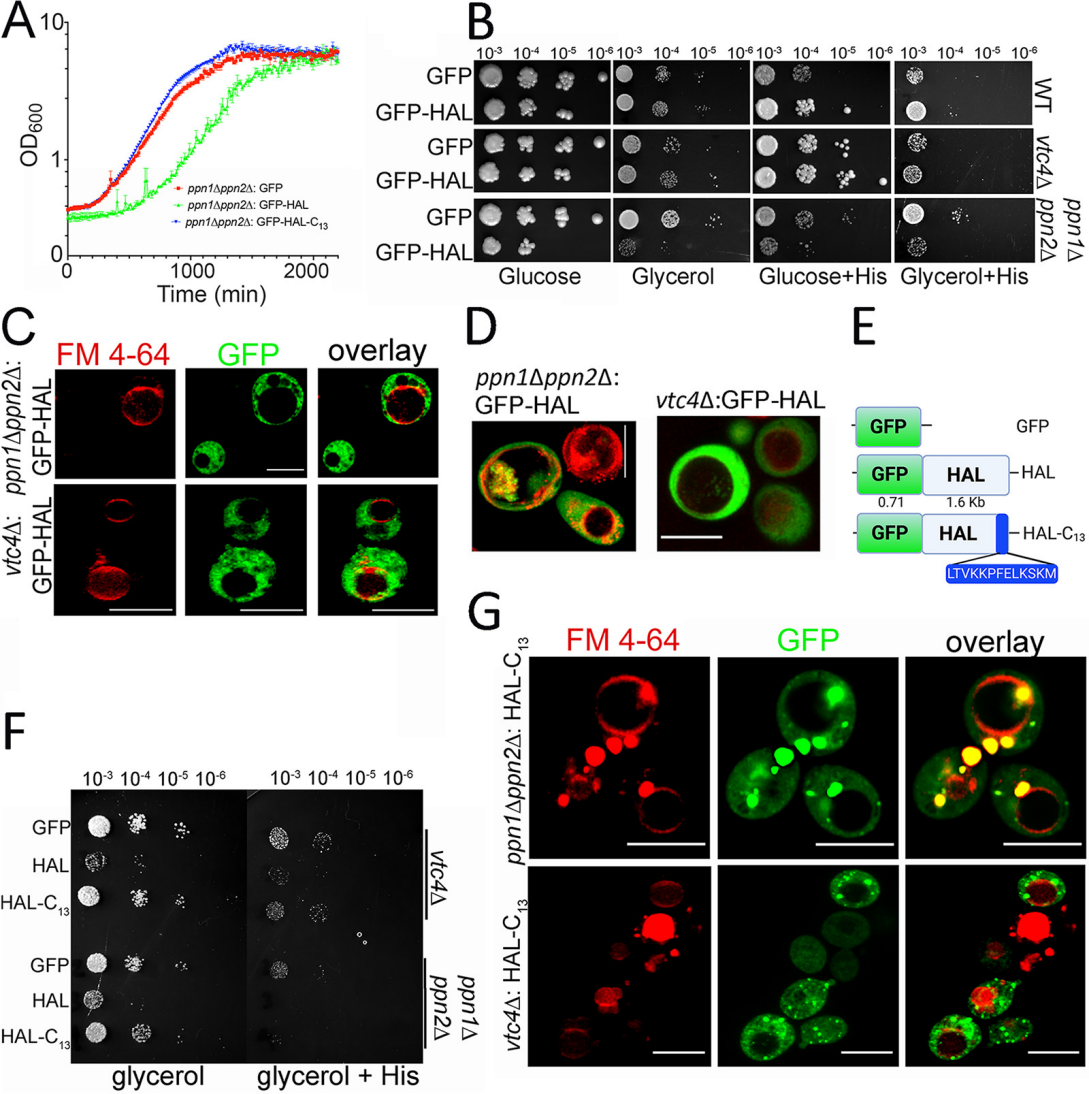
Expression of HAL in yeast. (A) Growth curve of HAL-expressing cells was established in the three mutants derived from the *ppn1*Δ *ppn2*Δ background strain transformed with GFP alone (control; red), with GFP-HAL (green), or with mutated HAL-C_13_ (blue). Cells were adjusted to the same densities (OD_600_ = 0.05) in 2 ml of SC Ura^−^ glucose liquid media. Growth was recorded spectrophotometrically for 34 h, and values (means ± SD absorbance values [*n* = 3]) converted to logarithmic function. (B) Spot growth assays of S. cerevisiae mutants grown in SC Ura^−^ solid medium with 2% glucose (wt/vol) or 2% glycerol (vol/vol) and supplemented with 2.5 mM l-histidine. The parental cell line DDY1810 (WT) and *vtc4*Δ and *ppn1*Δ *ppn2*Δ mutants were transformed with GFP (control) or GFP-HAL constructs. Cells were adjusted to an *A*_600_ of 0.1, and four serial dilutions were applied to petri dishes. Colony formation was recorded after 72 h or 96 h of growth at 30°C in glucose or glycerol medium, respectively. (C) Fluorescence microcopy of yeast cells harvested at the exponential phase of growth. Incorporation of FM4-64 dye in the vacuolar membrane was analyzed in live cells that constitutively express HAL fused to GFP. Bar = 5 μm. (D) Fluorescence microscopy of HAL-expressing yeast cells collected at the stationary phase (OD_600_ = 2) of growth in SD Ura^−^ Glc. Only overlaid channels are shown. Bar = 5 μm. (E) A yeast cell line lacking the last 13 residues of HAL (blue box) was generated (HAL-C_13_). (F) Drop tests were performed to compare growth fitness of yeast strains plated on SD Ura^−^ glycerol 2% (vol/vol) (glycerol) or SD Ura^−^ glycerol with 2.5 mM l-histidine (His) after 5 days. (G) Fluorescence microscopy of yeast cells expressing the GFP-HAL-C_13_ product in the polyP mutant strains. Cells were collected at exponential phase of growth. Incorporation of FM4-64 dye in the vacuolar membrane was analyzed in live cells that constitutively express HAL fused to GFP. Bars = 5 μm.

The budding yeast is the most common model for studying polyP metabolism. Therefore, we investigated the function of HAL in yeast. We expressed GFP-HAL in Saccharomyces cerevisiae wild type (WT) or *vtc4*Δ (no polyP) or in yeast where both vacuolar endo-polyphosphatases were mutated, the *ppn1*Δ *ppn2*Δ mutant(higher level of long-chain polyP) (Fig. S2). While the expression of GFP-HAL did not affect the growth rate of the wild type (WT) or the *vtc4*Δ strain, we noticed that in the *ppn1*Δ *ppn2*Δ background, the growth in liquid culture (Sabouraud dextrose [SD] Ura^−^ medium) was affected (Fig. 7A). This effect was greater when the carbon source was changed to the nonfermentative substrate glycerol (Fig. 7B), which has a direct effect on intracellular pH (pH_i_) regulation in yeast, as these cells are deficient in the assembly of the vacuolar H^+^-ATPase (19). To analyze whether exogenous addition of histidine would result in a more prominent defect in growth, this amino acid was added in excess to yeast cells (5 mM). Yeast cells are capable of biosynthesizing histidine, but the histidine conversion pathway is absent. Expression of HAL had a detrimental effect in the *ppn1*Δ *ppn2*Δ double mutant grown in the presence of glucose (Fig. 7B, glucose+His). This effect was also noticed when glycerol was the carbon source, where the overall efficiency of sustaining yeast growth was lower than in glucose medium. In this heterologous expression model, HAL localizes to the cytosol and is excluded from the FM 4-64-stained vacuole (Fig. 7C). When yeast cells were collected at the late exponential phase of growth, HAL staining showed a vacuolar distribution typical of autophagic vesicles (Fig. 7D) (20).

10.1128/mBio.01981-21.2 FIG S2 PolyP analysis in yeast mutants expressing HAL. (A) Total polyP extracted from GFP-HAL and GFP yeast strains and analyzed by PAGE and toluidine blue staining. (B) Equal amounts (50 μg) of total polyP were extracted from WT-background S. cerevisiae polyP mutants and resolved by PAGE followed by toluidine blue staining. PolyP_20_, polyP_100_ (2 mM), and inositol hexakisphosphate (IP_6_) were used as standard references to compare with samples as indicated above the gels. OG, orange G. Download FIG S2, TIF file, 2.5 MB. Copyright © 2021 Mantilla et al. 2021 Mantilla et al. https://creativecommons.org/licenses/by/4.0/ This content is distributed under the terms of the Creative Commons Attribution 4.0 International license.

We also analyzed the function of the basic C-terminal region of HAL (Fig. 7E) in yeast. Western blot analysis showed the expression of these proteins (Fig. S1A). Transformation of yeast mutants with a modified version of HAL lacking these last 13 residues did not affect the yeast growth in either liquid media (Fig. 7A) or plates with added glycerol (Fig. 7F). However, these yeasts also displayed abnormal localization of HAL, which remained accumulated in large vesicles, probably of endocytic origin, given their staining by FM 4-64 (Fig. 7G).

The analysis of polyP content in HAL-transformed yeast strains showed no marked differences with the WT control (Fig. S2A). These data suggest that the absence of polyP hydrolysis capacity along with histidine deamination may trigger autophagic events affecting yeast growth and that the lysine-rich C-terminal domain is essential for this phenotype.

## DISCUSSION

Alkalinization of acidocalcisomes is important for polyP hydrolysis and Ca^2+^ release from these organelles (6), but a physiological mechanism for this process had not been identified until now. In this work, we demonstrate that histidine is involved in the *in vitro* and *in vivo* alkalinization of the acidocalcisomes of T. cruzi epimastigotes due to the presence of HAL activity. We also found evidence that HAL interacts with polyP, not by polyphosphorylation but via electrostatic interactions with a C-terminal basic domain of the protein, supporting the finding that HAL is a polyP-binding protein (10). Deletion of this C-terminal basic region results in less alkalinization by histidine in permeabilized cells and in the parasites’ demise under starvation conditions. Deletion of this region also prevented the toxic effect of HAL when expressed in yeast mutants deficient in endopolyphosphatases, revealing the requirement of this basic region for polyP binding.

The presence of HAL in acidocalcisomes of T. cruzi is consistent with the finding that cationic amino acids can be transported and accumulated within these stores (7, 21). This process is conserved in yeast, where histidine mostly accumulates inside the vacuole through action of the *vba1* permease (22). More importantly, once incorporated into acidocalcisomes, histidine can be deaminated, producing ammonia. This conversion is possible even at the acidic pH found in these stores, where the imidazole side chain can be protonated (pK_a_, 6.0) but not the alpha amino group (pK_a_, 9.17). Release of NH_3_ within these organelles can also counterbalance the high concentration of protons taken up by the action of V-PPase and V-ATPase pumps (1). Although an NH_3_-driven alkalinization was previously observed within these organelles when parasites underwent hypo-osmotic stress (8), the cellular trigger had not yet been defined.

Histidine is abundant in the triatomine bug’s excreta (23). In T. cruzi, histidine is actively taken up by insect forms, and its metabolism yields CO_2_ (17). As HAL expression has been shown to be upregulated in epimastigotes and metacyclic trypomastigotes but repressed in intracellular amastigotes (11), it is reasonable to hypothesize that an increased histidine deamination capacity would be detrimental to intracellular development. Once T. cruzi enters the host cell, trypomastigote-containing vacuoles fuse with lysosomes, forming the parasitophorous vacuole (PV), and only when parasites egress to the cytosol can they divide as amastigotes. Egress from the PV is strongly inhibited by pharmacological treatment with chloroquine or NH_4_Cl, leading to a rise in the PV luminal pH (24). This effect is also consistent with the inability of the parasite to leave the PV when glutamine synthase (which transfers NH_3_ to glutamate) is chemically inhibited (25). The toxic effect caused by histidine-dependent intracellular NH_3_ formation was evident in our assays with live epimastigotes expressing HAL-pH.

Acidocalcisomes contain large amounts of polyP, which was previously found to interact with HAL (10). We showed that polyP inhibits HAL activity, thus suggesting that polyP has a modulatory role inside these acidic stores and that it binds to the C-terminal basic region of the enzyme. The importance of this C-terminal basic region is also evident in HAL-overexpressing yeast devoid of endopolyphosphatase activities. While these cells show a defect in growth, which is enhanced by histidine addition, the growth defect is undetectable when HAL is expressed with a deletion in the C-terminal region. The growth defect phenotype observed in yeast expressing HAL was exacerbated when the carbon source was switched from glucose to the nonfermentable one glycerol. Glycerol can sustain growth of yeast cells, but their capacity to regulate cytosolic pH by pumping H^+^ inside the vacuole is limited by the dysfunction of the V-ATPase under glucose depletion (19). Interestingly, polyP_3_, which is highly abundant in trypanosomes (26), has been suggested as a metabolite produced to counterbalance pH stress provoked by alkalinization in yeast and algae, where a rise in vacuolar pH activates polyP phosphatases (27, 28). Importantly, although polyP can also form a complex with cationic amino acids (such as arginine, lysine, and histidine) inside acidic vacuoles, this chemical interaction would not be sufficient as the mechanism to counterbalance the effects driven by histidine (7, 29). In summary, our results revealed an important role of histidine metabolism in regulating the acidocalcisome pH.

## MATERIALS AND METHODS

### Chemicals and reagents.

Blasticidin hydrochloride (10 mg/ml stock solution), hygromycin B (50 mg/ml solution), Geneticin sulfate (75% [wt/vol] purity), SuperSignal West Pico Plus chemiluminescent substrate, 6×His tag monoclonal antibody horseradish peroxidase (His.H8), and mouse antihemagglutinin (anti-HA) monoclonal antibody were from Invitrogen (Thermo Fisher Scientific). l-Histidine, anti-α-tubulin monoclonal antibody, mouse anti-c-Myc, ammonia assay kit, acid-washed glass beads (425 to 600 μm), protease inhibitor cocktail (catalog no. P2815), resazurin, EDAC [1-ethyl-3-(dimethylaminopropyl)carbodiimide] HCl, and rabbit anti-FLAG were from Sigma Chemical Co., and chicken anti-GFP was from Abcam. Guinea pig anti-vacuolar proton pyrophosphatase (VP1) was produced in-house (30), rabbit anti-pyruvate phosphate dikinase (PPDK) was a gift from Frédéric Bringaud (University of Bordeaux, France), mouse anti-histidine ammonia lyase (HAL) was kindly provided by Ariel Silber (University of São Paulo, Brazil). Aminomethylenediphosphonate (AMDP) was a gift from Eric Oldfield (University of Illinois at Urbana-Champaign), polyP_100_ (i.e., 100 P_i_ units) was kindly provided by Shiba Toshikazu (RegeneTiss Co., Japan) or was purchased from Kerafast. Polymethacrylate beads (ReliZyme EA113; particle size, S, EC-HA) carrying an ethylenediamino group were purchased from Resindion srl (Milan, Italy).

### Parasite culture.

T. cruzi epimastigotes (Y strain; discrete typed unit Tc-II) were maintained at 28°C in axenic cultures by successive passages in liver infusion tryptose (LIT) medium (31) supplemented with 10% heat-inactivated fetal bovine serum (FBS) and 2 mg/ml of hemin. Epimastigotes were differentiated into metacyclic trypomastigotes after transferring stationary-phase epimastigotes (∼2 × 10^7^ parasites/ml) into triatomine artificial urine (TAU-3AAG; 190 mM NaCl, 8 mM potassium phosphate buffer [pH 6.0], 17 mM KCl, 2 mM CaCl_2_, 2 mM MgCl_2_, 50 mM sodium glutamate, 10 mM l-proline, 2 mM sodium aspartate, and 10 mM dextrose) as previously described (18). To compare cell viability in a histidine-dependent medium, we used TAU-histidine, where 10 mM l-histidine was added as the sole carbon source. The transgenic epimastigotes transformed with the HAL-pH cassette were maintained in the presence of 75 μg/ml Geneticin (G418). Genome-edited parasites using a CRISPR-Cas9 system were maintained with 0.25 mg/ml of each drug, G418 and hygromycin (HYG), for endogenous C-terminal tagging.

### CRISPR/Cas9-mediated endogenous tagging.

To study the function of histidine ammonia-lyase (TcYC6_0121440; 1,605 bp), we applied CRISPR Cas9-based methods to endogenously attach fusion tags (3×HA and 3×c-Myc) to its C terminus. For this aim, the 3′ extremity of *HAL* was targeted by sgRNA to attach an epitope tag and induce homology-directed repair with donor templates containing normal HAL (C1) or C-end-mutated versions of HAL (C2). Point K-L substitutions at the residues K525, K526, K531, and K533 were introduced in the forward ultramer used to amplify donor DNA for C2-tagged parasites (primers 4 and 5 or primers 5 and 6) (Table S1). A specific single guide RNA (sgRNA) was cloned into Cas9/pTREX-*neomycin*, resulting in HAL_C-sgRNA/Cas9/pTREX-*n*. The donor templates contained a hygromycin resistance gene (*hph*) used as a selection marker.

10.1128/mBio.01981-21.3 TABLE S1 Oligonucleotides used for genome editing and cloning procedures for HAL-pH, yeast assays, and mutagenesis. Download Table S1, PDF file, 0.10 MB. Copyright © 2021 Mantilla et al. 2021 Mantilla et al. https://creativecommons.org/licenses/by/4.0/ This content is distributed under the terms of the Creative Commons Attribution 4.0 International license.

Donor DNA templates for homologous recombination were amplified by PCR with 120-nucleotide (nt) ultramers (primers 4 and 5) (Table S1), of which 100 nt corresponded to regions located immediately upstream of the stop codon and downstream of the Cas9 3′ cleavage site of HAL and 20 nt was for annealing with consensus regions present in the vectors pMoTag-HX1-4H for 3×HA or pMoTag-23M for 3×Myc terminal tagging used as templates in PCRs (32). Specific oligonucleotides were used in PCRs with Q5 high-efficiency DNA polymerase and 20 ng of plasmid DNA as the template. PCRs were amplified using Q5 high-fidelity DNA polymerase 2× master mix (New England Biolabs [NEB], MA, USA), 25 pmol of specific ultramers, and 20 ng of DNA template. PCR cycling conditions were initial denaturation for 2 min at 98°C, followed by 35 cycles of 20 s at 98°C, 10 s at 60°C, and 1 min at 72°C followed by a final extension for 5 min at 72°C. DNAs were precipitated with sodium acetate and cold ethanol and resuspended in nuclease-free water to be used in transfections. Then, 25 μg of HAL_C-sgRNA/Cas9/pTREX-*n* construct and donor templates (C1 and C2) was cotransfected to epimastigote forms and cultured for 5 weeks in the presence of G418 plus hygromycin (250 μg/ml each).

After the selection process, clonal populations were obtained by limiting dilution in conditioned medium (LIT–20% FBS plus selection antibiotics) and then seeded at 0.5 parasite/well in a 96-well plate under a humid atmosphere. Genetic validation was performed by PCR using specific primers that anneal to the 3′ extremities of the *HAL* coding sequence (CDS) and/or the selectable *hph* gene (primers 7 and 8) (Table S1). To verify the insertion of mutations in the HAL knock-in cell lines, sequencing reactions were performed from PCR amplicons using primer 7 (Table S1).

### Cloning procedures.

To generate C-terminally tagged cell lines for HAL, gene-specific single guide RNA (sgRNAs) to induce a double-strand DNA break inside the 3′ region of *HAL* CDS (protospacer-adjacent motif [PAM] sequence, ATT at position +1607) were amplified by PCR using specific primers (primers 1 and 2) (Table S1) and cloned into the BamHI site of the Cas9/pTREX-*n* vector. Cloning was performed by standard procedures using T4 DNA ligase (Promega) and Antarctic phosphatase (NEB) treatment. The amplification conditions were the same as described previously, and confirmation was performed by DNA sequencing to yield the HAL_C-sgRNA/Cas9/pTREX-*n* construct (32).

In order to engineer a genetically encoded pH sensor that enables spatiotemporal recording on live trypanosomes, we fused the *HAL* CDS to a pH modular cassette containing the DsRed and pHluorin reporter genes arranged in tandem. This pH module (DsRed-pHluorin) was excised from plasmid pAS1NB (33) using XbaI and NotI restriction sites and cloned into the vector pTREX-*omni* (34) to yield FLAG-DsRed-pHluorin/pTREX-*n*. Then, the HAL open reading frame was amplified by PCR (primers 9 and 10) (Table S1) and cloned into the HindIII and SalI sites of FLAG-DsRed-pHluorin/pTREX-*n* plasmid using the Gibson assembly master mix (NEB) to produce the FLAG-HAL-DsRed-pHluorin/pTREX-*n* construct. The construct was sequenced using a specific HAL primer (Table S1) (primer 7) to confirm that HAL was fused in frame to the pH module.

For yeast work, the full-length HAL CDS was cloned in frame with the 3′ extremity of eGFP gene sequence. HAL fragment was amplified by PCR and cloned into SalI-NotI sites of pCA58 (35) by Gibson reaction (primers 11 and 12) (Table S1) to yield the *GFP-HAL*/pCA58 construct. A mutated version of HAL lacking the last 13 residues located at its C terminus (HAL-C_13d_) was generated and introduced into yeast mutants. For this, specific primers (primers 13 and 16) (Table S1) were designed to introduce a stop codon at position V521 using 2 ng of the *GFP-HAL*/pCA58 construct as the template and following the procedures in the Q5 site-directed mutagenesis kit (NEB). Cloning was confirmed by sequencing using a HAL-specific primer (primer 7 or 14).

### Purification of recombinant HAL.

Recombinant HAL was produced in bacteria and purified using immobilized metal affinity chromatography and expression conditions described previously (36). Production of recombinant 6×His-HAL was done using Escherichia coli BL21(DE3) as the host strain transformed with construct pET28/HAL (kindly provided by Ariel Silber, USP, Brazil). Bacterial cells were grown in LB-kanamycin broth with 0.5 mM IPTG (isopropyl-β-d-thiogalactopyranoside) (optical density at 600 nm [OD_600_] = 0.5) for 4 h at 28°C with constant stirring (180 rpm). Purification of recombinant products was performed by immobilized metal affinity chromatography (IMAC) using 1 ml HisPur nickel-nitrilotriacetic acid (Ni-NTA) cartridges (Thermo Fisher) coupled to an AKTA Prime Plus fast protein liquid chromatography (FPLC) purification system. Protein was dialyzed against buffer (20 mM Tris-HCl [pH 8.0], 150 mM NaCl, 50 mM imidazole, 10% glycerol [vol/vol]) and concentrated by centrifugation (2,000 × *g* for 40 min at 4°C) using a 50-kDa-molecular-weight-cutoff (MWCO) filter concentrator (Merck Millipore). A mutated version lacking the last 13 amino acids found in the C terminus (6×His-HAL-C_13_) was produced by site-directed mutagenesis. HAL-specific primers (primers 15 and 16) (Table S1) and 2 ng of the pET28/HAL construct were used in an amplification reaction following manufacturer’s indications (NEB). Expression conditions were the same as detailed above for 6×His-HAL. Protein was quantified by BCA assay and used for binding assays as detailed below.

### Activity assay for HAL.

HAL activity was monitored spectrophotometrically by following absorbance changes of urocanate (*A*_277_) formed from histidine deamination (37). Protocol was adapted to 96-well microplates in a final volume of 0.21 ml, and kinetics were determined at 30°C for 10 min. The reaction buffer contained 100 mM Tris-HCl (pH 8.8), 0.1 mM MnCl_2_, and 1.7 mM reduced glutathione, and epimastigote homogenates (150 μg or 10 μg; 793 nM; *M*_r_, 59.5 kDa) of recombinant enzyme were used as the enzyme source. Kinetic parameters were determined by calculating the initial velocities as a function of the concentration of l-histidine (0.2 to 5 mM). Four catalytical sites were considered.

For assays in the presence of polyP, we detected changes in the fluorescence (excitation, 266 nm; emission, 337 nm; 5-nm slit width) of urocanate in quartz cuvettes (1 cm^2^) with constant stirring (38). For this, HAL was preincubated at room temperature with various concentrations (0.1 to 2 μM) of polyP_130_ for 2 min followed by addition of 5 mM l-histidine to start the reaction. In this case, kinetics was followed up for 5 min.

### Parasite transfection and cell sorting.

Transfection of epimastigotes was performed using 1 × 10^7^ cells per transformation collected at log phase of growth (<1.2 × 10^7^ parasites/ml). Cells were centrifuged at 1,000 × *g* for 10 min, washed with 15 ml of ice-cold sterile buffer A with glucose (BAG; 116 mM NaCl, 5.4 mM KCl, 0.8 mM MgSO_4_, 50 mM HEPES-KOH [pH 7.2], and 5.5 mM dextrose), centrifuged at 1,000 × *g* for 7 min, resuspended in 0.4 ml cytomix buffer (2 mM EGTA, 3 mM MgCl_2_, 120 mM KCl, 0.5% glucose wt/vol, 0.15 mM CaCl_2_, 0.1 mg/ml bovine serum albumin, 10 mM K_2_HPO_4_/KH_2_PO_4_, 1 mM hypoxanthine and 25 mM HEPES-Na, adjusted to pH 7.6), and transferred to an ice-cold 4-mm-gap cuvette (Bio-Rad) containing 10 μg of plasmid DNA from *pH-HAL*/pTREX-*n*. Cuvettes were chilled 10 min on ice, and three pulses of electroporation in a Bio-Rad Gene Pulser XCell electroporation system at 1.5 kV and 25 μF were applied as established for T. cruzi (39). The cell mixture was transferred to LIT–15% fetal calf serum (FCS), and 250 μg/ml of G418 was added to the cultures after 16 h of electroporation. Stable cell lines were obtained after 2 weeks under drug selection. Following this, we enriched our cultures with the MoFlo Astrios EQ cell sorter (Beckman Coulter). The instrument was calibrated in the green and red channels, and doubly positive (561-614/20 mCherry and 488-513/26 GFP) parasites corresponding to a total of 14,000 events were collected in fresh medium supplemented with 20% FCS. This cell line was termed pH-HAL.

### Protein electrophoresis and immunoblotting assays.

Protein electrophoresis was performed following the method described by Laemmli under reducing conditions (40). For immunoblotting in T. cruzi, total protein homogenates were prepared from parasites lysed in radioimmunoprecipitation assay (RIPA) buffer (150 mM NaCl, 20 mM Tris-HCl [pH 7.5], 1 mM EDTA, 1% SDS, and 0.1% Triton X-100 with 1× cOmplete EDTA-free protease inhibitor mixture and 1 mM phenylmethylsulfonyl fluoride [PMSF] added). Lysates were kept on ice for 30 min, passed through a 1-ml syringe (29 gauge), and then centrifuged at 12,000 × *g* for 30 min at 4°C. Supernatants were used in protein quantification using a bicinchoninic acid kit (Thermo Fisher Scientific), where equal amounts (50 μg) from each sample were separated by SDS-PAGE. Electrophoresed proteins were transferred to polyvinylidene difluoride (PVDF) membranes using the mini-Trans Blot cell system (Bio-Rad). Following transfer, membrane blots were blocked using Odyssey blocking solution (Li-Cor, Biosciences) for 1 h at RT. Blots were probed with appropriate antibodies as detailed in the figure legends. Antibody solutions were diluted in 5% skim milk (wt/vol) dissolved in phosphate-buffered saline (PBS) containing 0.3% Tween 20 (PBST) as follows: mouse anti-HA tag monoclonal antibody (1:2,000), anti-α-tubulin monoclonal antibody (1:45,000), mouse anti-c-Myc (1:1,000), rabbit anti-GFP, anti-FLAG (1:2,000), and polyclonal anti-HAL (1:5,000) for 2 h at room temperature (RT) under slow agitation. After washing three times with PBST, the blots were incubated with IRDye 800CW goat anti-mouse IgG and IRDye 680LT goat anti-rabbit IgG (Li-Cor Systems) diluted in PBST (1:10,000). Detection was performed using an Odyssey CLx imaging system (Li-Cor, Biosciences) with the appropriate filters. For the polyphosphate-mediated electrophoretic shift assay, proteins were resolved on 4 to 20% NuPAGE gels (Invitrogen), electrotransferred to PVDF membranes, and probed against anti-GFP antibodies. For chemiluminescent detection, membranes were incubated with enhanced chemiluminescence (ECL) substrate and signal developed using a ChemiDoc MP imaging system (Bio-Rad) or a film developer.

### Immunofluorescence microscopy.

Wild-type and endogenously tagged epimastigotes were washed with BAG and fixed with 4% paraformaldehyde in PBS (vol/vol) for 1 h, at RT. Cells were adhered to polylysine-coated coverslips and then permeabilized for 5 min with 0.3% Triton X-100. After permeabilization, cells were blocked with PBS containing 3% bovine serum albumin (BSA) (wt/vol), 1% fish gelatin (vol/vol), 50 mM NH_4_Cl, and 5% goat serum for 1 h at RT. Samples were incubated with primary antibody solutions containing one of these antibodies: monoclonal antibody (MAb) anti-Myc (1:50), mouse anti-HA (1:150), mouse polyclonal serum anti-HAL (1:200) or guinea pig anti-VP1 (1:250), diluted in PBS (pH 7.4) with 1% BSA, and then incubated for 1 h at RT. Cells were washed five times with PBS (pH 8.0) and then incubated for 1 h at RT (light protected) with Alexa Fluor 488-conjugated goat anti-mouse immunoglobulin, Alexa Fluor 488-conjugated goat anti-guinea pig IgG (heavy plus light chain [H+L]) or Alexa Fluor 546-conjugated goat anti-mouse antibodies (1:1,000). Then, samples were washed three times and mounted on slides using Fluoromount-G mounting medium with 3 μg/ml of 4′,6-diamidino-2-phenylindole (DAPI) to stain DNA. As a control, WT cells were incubated solely with secondary-antibody solution. Differential interference contrast and fluorescence optical images were captured on a Delta Vision II inverted microscope system (Olympus IX-71) with a 100× objective. Z-series were acquired and deconvoluted using SoftWoRx software. Superresolution structured-illumination microscopy (SR-SIM) images were acquired using a Zeiss Elyra S1 microscope with a 100× objective. Z-series stacks were processed through the SR-SIM analysis module of ZEN 2011 software (Carl Zeiss AG, Jena Germany). Final panel images were produced using Fiji (41) and Adobe Photoshop software.

### Time-lapse recordings.

For live-cell imaging of parasites expressing the HAL-pH transgene, cells were grown to log phase (<1.25 × 10^7^ epimastigotes) in LIT–10% FCS medium with 50 μg/ml G418. Cells were harvested by centrifugation (1,000 × *g* for 8 min) and washed out in BAG buffer. Samples were resuspended in cold BAG buffer supplemented with 125 mM sucrose. In parallel, plastic 35 mm μ-Dishes (ibidi GmbH, Grafelfing, Germany) were treated with 1 ml of poly-l-lysine (MW, 150,000 to 300,000) sterile solution (0.1 mg/ml in PBS) and incubated for 20 min at RT. Excess was removed and washed out once with BAG. Cell suspensions (1 ml) were then added to the dishes and allowed to settle for 30 min on a foil-covered ice box. Excess was carefully removed with a micropipette and gently washed with 1 ml of PBS. Then, adhered cells were added of 0.47 ml of BAG sucrose and imaged straight away. Time-lapse recordings were acquired using a 63× oil immersion objective under the differential interference contrast (DIC), GFP, and tetramethyl rhodamine isocyanate (TRITC) detectors of a Delta Vision II inverted microscope system (Olympus IX-71). Laser gain was manually adjusted (<0.5) and focus optimized for each run. Substrate additions were made using 0.2-ml long tips from stock solutions dissolved in BAG plus sucrose. Final concentrations were 20 mM sodium propionate, 16 mM l-histidine (pH adjusted to ∼9 in BAG), and 23 mM ammonium chloride, as detailed in the figure legends. A record with control vehicle was done with the solution used to dissolve histidine.

### Subcellular fractionation of acidocalcisomes and enzyme assays.

Lysates from epimastigotes (2 × 10^9^ parasites grown in glass Erlenmeyer flasks with mild agitation) were fractionated using a high-density gradient of iodixanol to isolate acidocalcisomes, as previously described (42). The fraction containing acidocalcisomes was resuspended in intracellular buffer, and 0.1 ml was resuspended in 3-ml quartz cuvettes for acridine orange incorporation measurements, as detailed below. Additions were made as detailed in figure legends. To analyze the content of the obtained fractions, we performed immunoblotting detection using anti-HAL, anti-VP1, and anti-PPDK. A vacuolar H^+^-pyrophosphatase (PPase) assay was carried out by measuring the AMDP-sensitive P_i_ release activity in the isolated fractions. P_i_ release was quantified using a malachite green assay (43). Ammonia release in acidocalcisome-enriched vesicles was performed in the presence/absence of l-histidine using an ammonia detection kit following the manufacturer’s instructions (Sigma-Aldrich).

### Fluorescence measurements of vacuolar acidification.

Vacuolar proton-translocating PPase activity was measured by monitoring the changes in fluorescence of the acridine orange dye as previously described for T. cruzi (3). Pyrophosphate-driven AO uptake was analyzed in both permeabilized parasites and acidocalcisome-enriched fractions. For *in situ* measurements with epimastigotes, parasites were harvested by centrifugation (1,000 × *g* for 7 min) and washed out with BAG buffer. Cells were adjusted to 5 × 10^8^ cells/ml, and 0.1 ml cells was resuspended in IC buffer (IC is 65 mM KCl, 125 mM sucrose, 2 mM MgSO_4_, 10 mM K-HEPES, 50 μM EGTA, with final pH adjusted to 7.2) under constant agitation at 30°C. Cell suspension was loaded with 3 μM acridine orange and 0.1 mM potassium pyrophosphate. Digitonin was added to a final concentration of 17 μM when used for intact cells, and further additions of 5 mM l-histidine (dissolved in PBS, pH adjusted to pH ∼9), l-glutamate-Na, or 1 μM ionomycin were made as stated in the figure legends.

### Parasite viability assay under differentiation medium.

To determine the role of histidine in HAL-C-terminal mutations, control and epitope-tagged epimastigotes were grown in LIT–10% FCS medium at logarithmic phase (48 h) and then transferred to selective triatomine artificial urine medium (TAU; 190 mM NaCl, 8 mM K-phosphate buffer [pH 6.0], 17 mM KCl, 2 mM CaCl_2_, and 2 mM MgCl_2_) supplemented with 10 mM l-proline, 50 mM Na-l-glutamate, 2 mM l-aspartate, and 10 mM dextrose (TAU-3AAG) (44) or with 10 mM l-histidine (TAU-H) as the sole carbon source. After 72 h of incubation at 28°C, cells were harvested, and viability was determined with an alamarBlue assay (45). For this, parasites were resuspended in PBS-glucose, added (1 × 10^6^ cells/well) to 96-well microplates in the presence of 54 μM resazurin (0.54 mM stock solution with 12.5 mg of resazurin in 100 ml of sterile PBS), and incubated for 4 h at 28°C under light protection. Changes in fluorescence (excitation at 544 nm, emission at 590 nm) were recorded and compared to control treatments.

### Yeast strains and culture conditions.

Saccharomyces cerevisiae mutant strains used in our study are isogenic to DDY1810 parental strain (WT) (46). The vacuolar transporter chaperone 4 (*vtc4*Δ)-null mutant, which is deficient in vacuolar polyP synthesis, and the double mutant strain lacking both vacuolar endopolyphosphatase PPN1 and metallophosphatase PPN2 activities (*ppn1*Δ *ppn2*Δ) were transformed with trypanosome HAL (the list of strains generated is presented in Table S2). Yeast transformation was performed with approximately 250 ng of the *GFP-HAL*/pCA58 expression construct or *GFP*/pCA58 (used as a control) using the lithium chloride standard method (47). pCA58 vector reverts the auxotrophy for uracil by complementing the function of *URA3* gene. Yeast transformants were recovered in synthetic complete medium plates containing 2% glucose, 2% agar base, yeast nitrogen base without amino acids (Sigma), and synthetic complete medium without uracil (SC Ura^−^ Glc) as previously described (14) or replaced by a nonfermentable carbon source, 3% glycerol plus 2% ethanol (SC Ura^−^ Glyc).

10.1128/mBio.01981-21.4 TABLE S2 Yeast strains generated in this study. Download Table S2, PDF file, 0.1 MB. Copyright © 2021 Mantilla et al. 2021 Mantilla et al. https://creativecommons.org/licenses/by/4.0/ This content is distributed under the terms of the Creative Commons Attribution 4.0 International license.

### Fluorescence microscopy.

To determine HAL localization in yeast, cells were grown overnight in 5 ml of SC Ura^−^ Glc at 30°C. For staining vacuole membrane, cells (OD_600_ of 0.2) were resuspended in 1 ml SC Glc medium containing 10 μM the acidogenic FM4-64 dye for 1 h at 30°C with constant shaking (150 rpm), as described previously (48). Cells were added to imaging cover glass chambers (Eppendorf, Germany) and visualized using an Apochromat 63× objective under immersion oil with the green (argon 488) and red (514) laser lines of a Zeiss LSM 880 Airyscan confocal microscope (Carl Zeiss, Germany). Images were processed as described under “Immunofluorescence microscopy” above.

### Growth assays.

Spot assays with yeast mutants herein generated were done to analyze cell growth. Briefly, cells were grown overnight in 5 ml SC Ura^−^ Glc and then four serial dilutions (dilution factor, 1/10) were made in 1 ml of sterile water starting at an OD_600_ of 0.1. After this, 2.5 μl of each cell suspension was added to plates with SC Ura^−^ 2% Glc, SC Ura^−^ 2% glycerol (vol/vol), or selective medium supplemented with 2.5 mM l-histidine. Plates were grown at 30°C for 3 or 5 days when glucose or glycerol, respectively, was used as a carbon source. Growth curves were performed in 12-well plates using cells pregrown overnight (∼14 h at 30°C) and diluted to an OD_600_ of 0.05 in 2 ml of the desired SC Ura^−^ medium. Changes in OD_600_ were monitored spectrophotometrically in a SPECTROstar Nano microplate reader (BMG Labtech, Offenburg, Germany) over 21 h at 30°C with orbital shaking.

### PolyP-dependent assays (polyP binding assay, polyP extraction, and electrophoresis shift assays).

Polyphosphate-20 (20 P_i_ units) was immobilized onto a solid support (polymethacrylate beads EC-HA) using EDAC (1-ethyl-3-[3-dimethylamino)propyl]carbodiimide) as a cross-linker agent by following the method described by Choi et al. (49). The yield obtained was 8.4 μg of polyP–20/mg (dry weight) of Sepabeads as determined by a malachite green assay after hydrolysis under acidic boiling. Affinity binding assay was performed in binding buffer (50 mM Tris-HCl [pH 7.4], 50 mM NaCl, 0.1% BSA) by incubating 10 μg of recombinant 6×His-HAL or 6×His-HAL-C_13_ produced in E. coli and approximately 200 μg immobilized polyP_20_.

Total polyphosphate was extracted from 50 ml of yeast cells grown in SC Ura^−^ glucose for 48 h at 30°C. Yeast pellets were lysed with LETS buffer (0.1 M LiCl, 10 mM EDTA, 10 mM Tris-HCl, pH 7.4, and 0.2% SDS) and glass beads (1/3 of total volume), and total polyP was extracted using acidic phenol and quantified as described previously (50). For electrophoresis shift assays, protein extracts were prepared from yeast cells grown overnight in 10 ml of fresh SC Ura^−^ Glc media. Proteins were extracted in ice-cold lysis buffer (50 mM Tris-HCl [pH 8.0], 150 mM NaCl, 5 mM dithiothreitol, supplemented with 1× protease inhibitors and phosphatase inhibitor mixture [20 mM imidazole, 1 mM sodium fluoride, 1.15 mM sodium molybdate, and 1 mM sodium orthovanadate]), as described in reference 14. To analyze the effect of exogenous addition of polyP on electrophoretic mobility of HAL, total protein extracts from strains derived from *vtc4*Δ and *ppn1*Δ *ppn2*Δ mutants were prepared. Then, 20 μl of each protein extract (1 mg/ml) was incubated with 5 μl of 2 mM polyP_100_ for 20 min at 30°C. Samples were added to 4× SDS sample buffer, boiled, resolved by NuPAGE (Invitrogen), and immunoblotted with appropriate antibodies.

### PAGE.

High-acrylamide gel electrophoresis was used to resolve polyP extracted from yeast cells generated in this study (Table S2). Products were mixed with 1× orange loading dye buffer and resolved by PAGE using 35% acrylamide–bis-acrylamide 19:1 gels (18 by 24 cm) in Tris-borate-EDTA buffer. After being run (7 mA for ∼21 h at 4°C), gels were stained with toluidine blue (30 min) and destained with 20% methanol (MeOH)-H_2_O, as previously described (51).

### Statistical analysis.

All statistical analyses were performed using software GraphPad Prism v8. Bar plots present the standard errors of the means (SEM) iterated from at least three biological replicates. *P* values of <0.05 were considered to indicate significance. Depending on the experiment, specific statistical posttests and trials (*n*) were applied and are indicated in figure legends.

10.1128/mBio.01981-21.7 VIDEO S3 Glutamate/histidine-driven alkalinization after acidic pulse. Live imaging was recorded in parasites seeded onto polylysine-coated slides, and changes in fluorescence (DsRed and pHluorin green) were monitored after sequential additions of propionic acid (30 s) followed by l-glutamate (1 min 30 s) and l-histidine (3 min). The assay was followed for approximately 5 min, and the movie was generated using 6 fps at medium speed. Download Movie S3, MOV file, 15.2 MB. Copyright © 2021 Mantilla et al. 2021 Mantilla et al. https://creativecommons.org/licenses/by/4.0/ This content is distributed under the terms of the Creative Commons Attribution 4.0 International license.

10.1128/mBio.01981-21.8 VIDEO S4 Parasite burst after alkaline stress. Live imaging was recorded in parasites seeded onto polylysine coated slides, and changes in fluorescence (DsRed and pHluorin green) were monitored after sequential additions of NH_4_Cl (30 s) followed by l-histidine (1 min 30 s). The assay was followed for approximately 5 min, and the movie was generated using 6 fps at medium speed. Download Movie S4, MOV file, 14.3 MB. Copyright © 2021 Mantilla et al. 2021 Mantilla et al. https://creativecommons.org/licenses/by/4.0/ This content is distributed under the terms of the Creative Commons Attribution 4.0 International license.
