# Supplementary figures and images for: The Histidine Ammonia Lyase of Trypanosoma cruzi Is Involved in Acidocalcisome Alkalinization and Is Essential for Survival under Starvation Conditions
Source: mBio. 2021 Nov 2;12(6):e01981-21. doi: 10.1128/mBio.01981-21 (PMC8561398; doi:10.1128/mBio.01981-21)

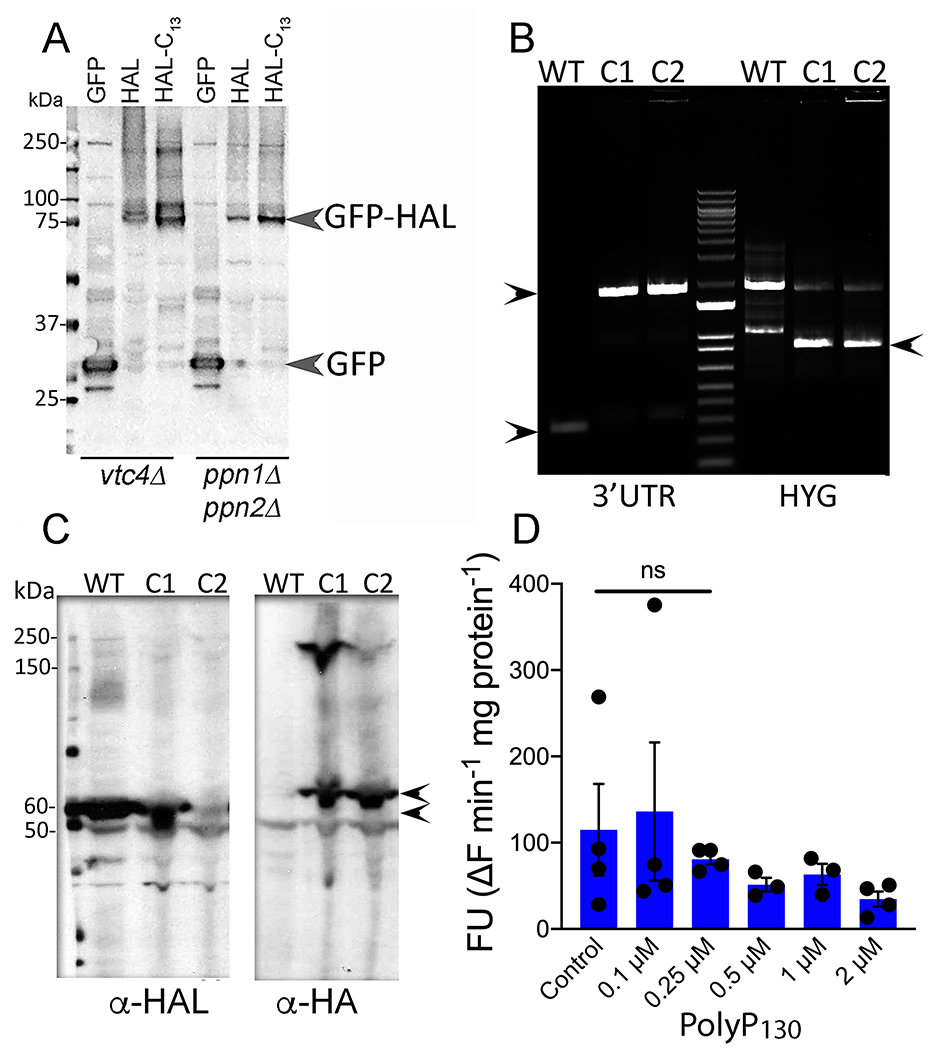

Supplement: FIG S1 [file mbio.01981-21-sf001.tif]

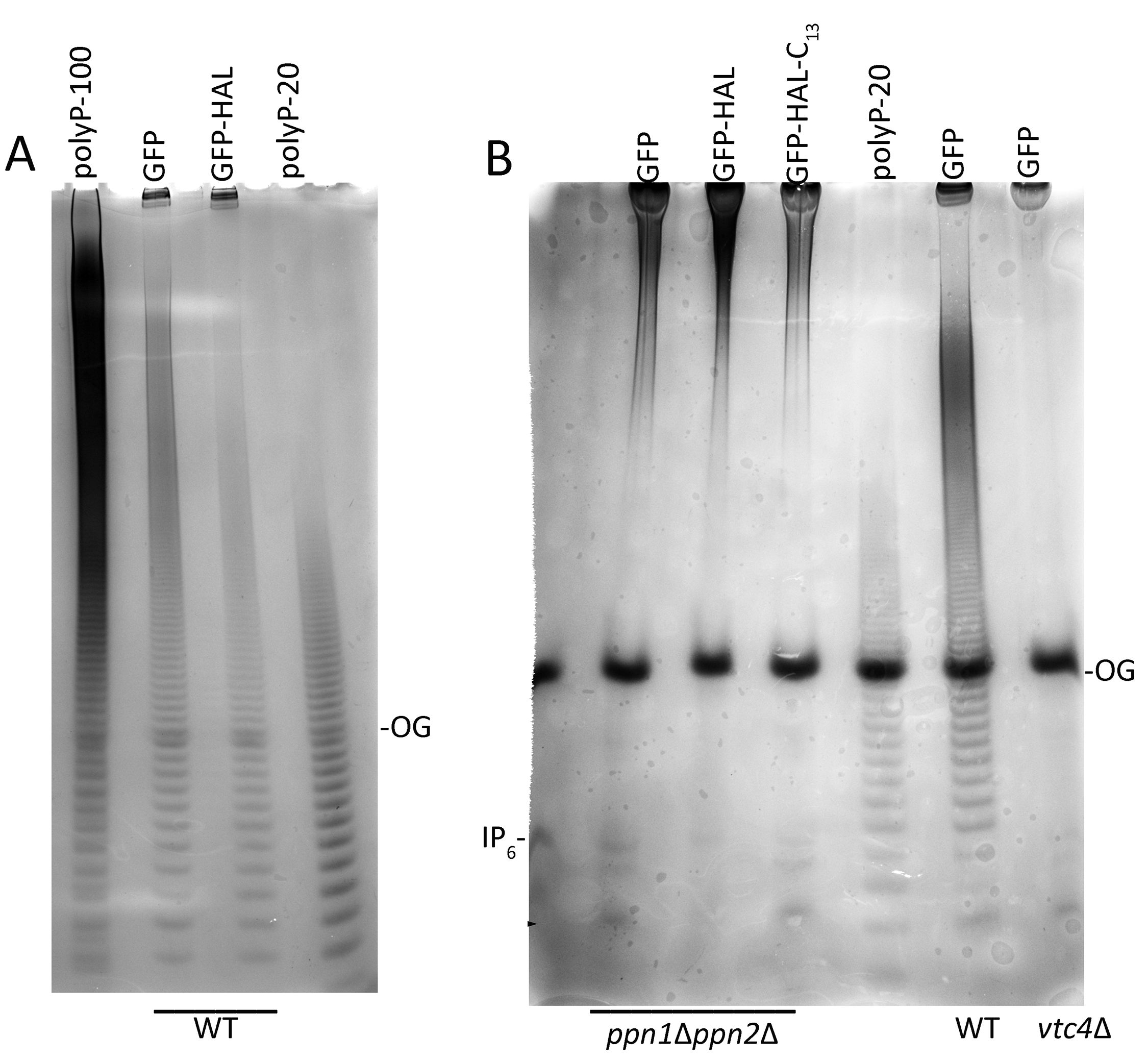

Supplement: FIG S2 [file mbio.01981-21-sf002.tif]
