# Supplementary material for: The Histidine Ammonia Lyase of Trypanosoma cruzi Is Involved in Acidocalcisome Alkalinization and Is Essential for Survival under Starvation Conditions
Source: mBio. 2021 Nov 2;12(6):e01981-21. doi: 10.1128/mBio.01981-21 (PMC8561398; doi:10.1128/mBio.01981-21)
Supplement: TABLE S1 [file mbio.01981-21-st001.pdf]

Sequence/origin	Nº	Name	Oligonucleotide sequence 5'-3'	Purpose
TcYC6_0121440	1	sgRNA-HAL_C	GATCGGATCCGCTGAAATCCAAGATGTGAAGTTTTAGAGCTAGAAATAGC	C-tagging by CRISPR Cas9
PMID: 27793988	2	sgRNA-scaffold_Rv	CAGTGGATCCAAAAAAGCACCGACTCGGTG	sgRNA amplification
PMID: 26199333	3	HX1_Rv	TAATTTCGCTTTCGTGCGTG	Verification of sgRNA cloning
TcYC6_0121440	4	HAL_HR1_C	TGCTGCGCTCCGGTGCAGTATGGAAAACCGTTAAGCCGTACGTCCCTGAGGAGGC GCGATTCTTGGGCGTCCTAACCGTTAAGAAACCATTTGAGCTGAAATCCAAGATGG GTACCGGGCCCCCCCTCGAG	Donor template amplification
TcYC6_0121440	5	HAL_HR2_C	CAGGAAAATCACGCTTCTACTCCAGTAAAGTCAAATTTTTTGACCAATATTATATA TATCGATATGGATGGATGAATATTTATATTGTGTTTGTTTGTGGCGGCCGCTCTAGA ACTAGTGGAT	Donor template amplification
TcYC6_0121440	6	HAL_HR1_C2	TATTGACAACGTCACCAAGCTGCTGCGCTCCGGTGCAGTATGGAAAACCGTTAAGC CGTACGTCCCTGAGGAGGCGCGATTCTTGGGCGTCCTAACCGTTCTGCTGCCATTT GAGCTGCTGTCCCTGATGGGTACCGGGCCCCCCCTCGAG	Donor template amplification
TcYC6_0121440	7	HAL_Ctag_1452	GACCGTGTCATCAACAC	Diagnostic PCR
TcYC6_0121440	8	HAL_Ctag_+138	CCATATGACTCTGCCACTTTCC	Diagnostic PCR
TcYC6_0121440	9	HAL-pH_Gib1	GGATCCGACGTCGAGCTCAAGCTTATGAGGGTTATCCTTGACGGC	Amplification of HAL-pH cassette
TcYC6_0121440	10	HAL-pH_Gib2	CATTTGAGCTGAAATCCAAGATGGGTGGCGACTAGTGGGGATCCCGGGCCCGCGG TACCGTCGAC	Amplification of HAL-pH cassette
TcYC6_0121440	11	HAL_pCA58_S	GATCCCCAGGAATTCCCGGGTCGACAAGGGTTATCCTTGACGGC	Amplification of HAL for yeast assays
TcYC6_0121440	12	HAL_pCA58_N	GGGCCCTCTAGACTCGAGCGGCCGCCATCTTGGATTTCAGCTCAAATGGTTTCTT	Amplification of HAL for yeast assays
TcYC6_0121440	13	HAL-C_{13}_ydel1	TGAGCGGCCGCTCGAGTC	Mutagenesis of HAL in yeast
PMID: 23861393	14	pCA58_eGFP-fw	GAGAGACCACATGGTCTTGTTAG	Sequencing in pCA58 vector
TcYC6_0121440	15	HAL_C13_del1	TGAGCTCGAGCACCACCAC	Mutation of recombinant 6His-HAL
TcYC6_0121440	16	HAL_C13_del2	GCCCAAGAATCGCGCCTC	Mutation of recombinant 6His-HAL

Table S1. Oligonucleotides used for genome editing, and cloning procedures for HAL-pH, yeast assays and mutagenesis.
