# Supplementary material for: The Histidine Ammonia Lyase of Trypanosoma cruzi Is Involved in Acidocalcisome Alkalinization and Is Essential for Survival under Starvation Conditions
Source: mBio. 2021 Nov 2;12(6):e01981-21. doi: 10.1128/mBio.01981-21 (PMC8561398; doi:10.1128/mBio.01981-21)
Supplement: TABLE S2 [file mbio.01981-21-st002.pdf]

Table S2. Yeast strains generated in this study

Strain	Background genotype	Vector	Reference
WT	DDY1810	-	PMID: 19614566
WT-gfp	DDY1810	pCA58- uracil-gfp	This study
WT-gfp::HAL	DDY1810	pCA58- uracil-gfp-HAL	This study
*vtc4*Δ	DDY1810 *vtc4*Δ	leucine	PMID: 25773596
*vtc4*Δ-gfp	DDY1810 *vtc4*Δ	pCA58- uracil-gfp	This study
*vtc4*Δ-gfp::HAL	DDY1810 *vtc4*Δ	pCA58- uracil-gfp:*HAL*	This study
*vtc4*Δ-gfp::HAL-C_{13}	DDY1810 *vtc4*Δ	pCA58- uracil-gfp:*HAL*-C_{13}	This study
*ppn1*Δ*ppn2*Δ	DDY1810 *ppn1*Δ*ppn2*Δ	pYM-natNT2, pYM-hphNT1	PMID: 31844018
*ppn1*Δ*ppn2*Δ-gfp	DDY1810 *ppn1*Δ*ppn2*Δ	pCA58- uracil-gfp	This study
*ppn1*Δ*ppn2*Δ-gfp::HAL	DDY1810 *ppn1*Δ*ppn2*Δ	pCA58- uracil-gfp:*HAL*	This study
*ppn1*Δ*ppn2*Δ-gfp::HAL-C_{13}	DDY1810 *ppn1*Δ*ppn2*Δ	pCA58- uracil-gfp:*HAL*-C_{13}	This study
